# Osteoprotegerin levels in ST-elevation myocardial infarction: Temporal profile and association with myocardial injury and left ventricular function

**DOI:** 10.1371/journal.pone.0173034

**Published:** 2017-03-02

**Authors:** Christian Shetelig, Shanmuganathan Limalanathan, Jan Eritsland, Pavel Hoffmann, Ingebjørg Seljeflot, Jon Michael Gran, Pål Aukrust, Thor Ueland, Geir Øystein Andersen

**Affiliations:** 1 Department of Cardiology, Oslo University Hospital Ullevål, Oslo, Norway; 2 Center for Clinical Heart Research, Oslo University Hospital Ullevål, Oslo, Norway; 3 Faculty of Medicine, University of Oslo, Oslo, Norway; 4 Feiring Heart Clinic, Feiring, Norway; 5 Center for Heart Failure Research, Oslo, Norway; 6 Section of Interventional Cardiology, Oslo University Hospital Ullevål, Oslo, Norway; 7 Oslo Center for Biostatistics and Epidemiology, Department of Biostatistics, Oslo University Hospital and University of Oslo, Oslo, Norway; 8 Research Institute of Internal Medicine, Oslo University Hospital Rikshospitalet, Oslo, Norway; 9 Section of Clinical Immunology and Infectious Diseases, Oslo University Hospital Rikshospitalet, Oslo, Norway; 10 K.G. Jebsen Inflammatory Research Center, University of Oslo, Oslo, Norway; Universita degli Studi Magna Graecia di Catanzaro, ITALY

## Abstract

**Background:**

Elevated levels of osteoprotegerin (OPG) have been associated with adverse outcomes in ST-elevation myocardial infarction (STEMI). However, the role of OPG in myocardial injury and adverse remodeling in STEMI patients remains unclear. The aims of this observational cohort study were to evaluate: 1) the temporal profile of OPG during STEMI, 2) possible associations between OPG measured acutely and after 4 months, with infarct size, adverse left ventricular (LV) remodeling, microvascular obstruction (MVO) and myocardial salvage and 3) the effect of heparin administration on OPG levels.

**Methods:**

Blood samples were drawn repeatedly from 272 STEMI patients treated with primary percutaneous coronary intervention (PCI). Cardiac magnetic resonance imaging (CMR) was performed in the acute phase and after 4 months. The effect of heparin administration on OPG levels was studied in 20 patients referred to elective coronary angiography.

**Results:**

OPG levels measured acutely were significantly higher than Day 1 and during follow-up. OPG levels were correlated with age. No association was found between early OPG levels and CMR measurements at 4 months. Patients with >median OPG levels measured at Day 1 had larger final infarct size, lower LV ejection fraction (LVEF) at 4 months and higher frequency of MVO. There were no associations between OPG and change in end-diastolic volume or myocardial salvage. OPG remained associated with infarct size and LVEF after adjustment for relevant covariates, except peak troponin T and CRP. A 77% increase in OPG levels following heparin administration was found in patients undergoing elective coronary angiography.

**Conclusions:**

OPG was found to be associated with myocardial injury, but not with LV remodeling or myocardial salvage. The use of OPG as a biomarker in STEMI patients seems to be limited by a strong association with age, confounding effect of heparin administration, and little additive value to established biomarkers.

## Introduction

Osteoprotegerin (OPG), a glycoprotein in the tumor necrosis factor (TNF) receptor superfamily, acts as a decoy receptor for the receptor activator of nuclear factor-κB ligand (RANKL) and TNF-related apoptosis inducing ligand (TRAIL) [1, 2]. OPG is expressed in most human tissues and cells, including bone (osteoblasts), vascular smooth muscle cells, and endothelial cells [3, 4]. Increased levels of OPG have been associated with coronary calcium score and the development and severity of coronary artery disease (CAD) [5–9]. Moreover, OPG, as well as RANKL, are expressed within the failing myocardium in both experimental and clinical heart failure (HF), and strong immunostaining of these molecules has also been found within atherosclerotic carotid plaques as well as in thrombus material obtained at the site of plaque rupture during myocardial infarction (MI) [10]. In addition, OPG has been identified as an independent predictor of HF development and mortality in patients with acute coronary syndrome (ACS) [11, 12]. Elevated serum OPG has been reported in ST-elevation MI (STEMI) patients compared to patients with non-STEMI, unstable angina, stable CAD, and controls [11, 13]. We have previously shown, in a study of 199 STEMI patients, that OPG levels measured a median of 16.5 hours after percutaneous coronary intervention (PCI) were significantly associated with infarct size assessed after 3 months by SPECT imaging [14]. However, the results of more recent studies investigating OPG and infarct size in STEMI patients have been inconsistent [15–17].

In order to explore the possible role of OPG in the extent of myocardial damage, adverse remodeling, impaired microcirculation and salvage of the myocardium, in spite of acute reperfusion of MI, we aimed to elucidate in detail the role of OPG in STEMI. The objectives of the present study were therefore: 1) to establish a temporal profile of OPG in STEMI, 2) to examine the association of OPG with markers of adverse left ventricular (LV) remodeling in patients with STEMI, 3) to investigate possible associations of OPG measured during acute STEMI with final infarct size, microvascular obstruction (MVO) and myocardial salvage, 4) to study a possible association between OPG levels measured in a clinically stable situation late after STEMI and adverse LV remodeling, and 5) to elucidate the effect of heparin administration on OPG levels.

## Material and methods

### Ethics

The Postconditioning in ST-Elevation Myocardial Infarction (POSTEMI) trial was approved by the Regional Committee for Medical Research Ethics, South-East Norway, on July 30, 2008. The trial was initiated on January 12, 2009 with a pilot period including 20 patients and following evaluation of safety by the Data and Safety Monitoring Board the trial was registered at clinicaltrials.gov on June 16, 2009 (NCT00922675). All parts of the study were conducted in accordance with the ethical principles of the Declaration of Helsinki, and all patients gave written informed consent to participate in the study. The study protocol and supporting STROBE checklist are available as supporting information. The authors confirm that all ongoing and related trials for this intervention are registered.

A separate, study was conducted in order to elucidate a possible effect of heparin on OPG release. This sub-study was approved by the Regional Committee for Medical Research Ethics, South-East Norway. All patients gave written informed consent to participate in the study.

### Study population

The POSTEMI trial was a prospective, randomized, single-center, open-label clinical trial investigating the cardioprotective strategy ischemic postconditioning (iPost). The study design has previously been reported in detail [18, 19]. Briefly, the study population consisted of 272 patients with first-time STEMI and symptom duration <6 hours included between January 12, 2009, and August 25, 2012, at Oslo University Hospital Ullevål, Norway. Two reperfusion strategies, PCI with iPost or conventional PCI, were compared. Patients with inability to provide informed consent, previous MI, renal failure (serum creatinine >200 μmol/L), contraindications for cardiac magnetic resonance imaging (CMR), and clinically unstable patients (cardiac arrest, cardiogenic shock, pulmonary congestion, or hypotension) were excluded. Occlusion (Thrombolysis In Myocardial Infarction [TIMI] flow 0-1) of the infarct related artery and successful reperfusion after the first balloon inflation (TIMI flow 2-3) had to be demonstrated angiographically before 1:1 randomization to iPost or control group. Adjuvant STEMI treatment was given according to current guidelines [20]. The effect of iPost on the primary endpoint of the study, final infarct size measured by CMR after 4 months, was neutral [19].

#### Sub-study on a possible effect of heparin administration on OPG release

In order to elucidate a possible effect of heparin on OPG release, 20 patients (median age 68, 80% male) with suspected stable CAD undergoing elective coronary angiography were included between August 31 and September 15, 2015, at Oslo University Hospital Ullevål, Norway. Exclusion criteria were ongoing ACS or current treatment with warfarin or a non-Vitamin K oral anticoagulant. Blood samples for OPG were drawn prior to the angiography procedure (venous sample), from the arterial cannula before administration of unfractionated heparin (all patients received 5000 IU and additional bolus if PCI was performed), and at the end of the angiography procedure.

### Biochemical analyses

A prospective objective of the POSTEMI trial was to study specific biomarkers in relation to LV remodeling and function. Blood samples for biobanking and analyses of OPG were drawn before and immediately after the PCI procedure, at Day 1 (median 14.7 hours after PCI), and at 4-month follow-up. Blood was centrifuged after coagulation (within 1 hour) at 2,500*g* for 10 minutes and serum was stored at -80°C in multiple aliquots until analyses. Serum levels of OPG were quantified by enzyme immunoassay using matched antibodies from R&D Systems (Minneapolis, Minnesota, USA) as previously described and validated [21]. The intra-assay and inter-assay coefficients of variation (CV) were <10%. The sensitivity was calculated to be 15 pg/ml. C-reactive protein (CRP) was determined by routine laboratory high-sensitivity assay, peak CRP was defined as the maximum value measured during hospitalization. Levels of N-terminal pro-B-type natriuretic peptide (NT-proBNP) on admission were determined with Elecsys proBNP sandwich immunoassay on Elecsys 2010 (Roche Diagnostics). Serum cardiac-specific troponin T (TnT) was measured by electrochemiluminescence technology for quantitative measurement (Elecsys 2010, Roche, Mannheim, Germany). The peak TnT level was defined as the maximum value measured during hospitalization. Inter-assay and intra-assay CV% were <7% for all assays.

### CMR protocol and analysis

CMR imaging was performed at a median of 2 days after the index event in the acute phase and repeated after 4 months’ follow-up. The details regarding CMR protocol and analyses have been published in detail previously [22]. In brief, a 1.5 T scanner (Philips Intera, release 11 or Philips Achieva, release 3.2, Best, Netherlands) was used to obtain images, and image analyses were performed on an extended MR Work Space (Philips Medical Systems). Short axis images of LV were acquired for complete volume analysis including LV ejection fraction (LVEF). T2 weighted imaging was performed in the short axis plane to quantify the area at risk, defined as myocardium with a signal intensity (SI) of more than 2 standard deviations above the SI in remote non-infarcted myocardium. Late gadolinium enhancement (LGE) imaging was obtained 15 minutes after contrast injection (Gadolinium-DTPA 469 mg/ml, 0.15 mmol/kg, Magnevist, Schering AG, Germany), and two and four chamber long axis views and short axis views were assessed to determine infarct size. Myocardial salvage index (%) was calculated as follows: [(area at risk – infarct size at 4 months)/area at risk] x 100 [23–25]. MVO was assessed in late enhancement images, and defined as a dark area within the hyperintense area in the infarcted myocardium.

### Statistical analyses

OPG was analyzed both as a continuous variable and as a dichotomized variable. Analyses of the association between OPG dichotomized at each quartile and end-diastolic volume (EDV), infarct size, LVEF, and myocardial salvage index resulted in different cut-off values for the different outcome variables. The median value was chosen as cut-off for all outcome variables due to considerations of power, and to make interpretation of the results easier. Non-parametric tests were used for group comparisons, due to skewness in some of the analyzed variables. Mann Whitney U test was used for group analyses of continuous variables, while categorical variables were analyzed using Chi-square test. Wilcoxon signed rank test was used to compare OPG levels at different sampling points. Multivariable linear regression analyses were performed with final infarct size, LVEF, myocardial salvage index, and delta EDV (change from baseline to 4-month) as outcome variables, respectively. The following covariates were entered into the models based on either clinical relevance or an association with either OPG or the dependent variable with a p-value < 0.2: Age, gender, time from symptom onset to PCI, infarct localization (anterior MI vs inferior or posterior MI), treatment with ischemic postconditioning, peak TnT, peak CRP and NT-proBNP on admission. As a result of skewness, the following continuous variables were logarithmically transformed: OPG, Troponin T, CRP, and NT-proBNP. Pairwise deletion was used to handle missing data in multivariable analyses (sensitivity analyses were also performed using listwise deletion showing similar results). Due to the explorative nature of the study, no correction for multiple comparisons was performed. A p-value <0.05 was considered statistically significant. All statistical analyses were performed using IBM SPSS for Windows version 21.0.

## Results

### Temporal profile of OPG

**Fig 1:**
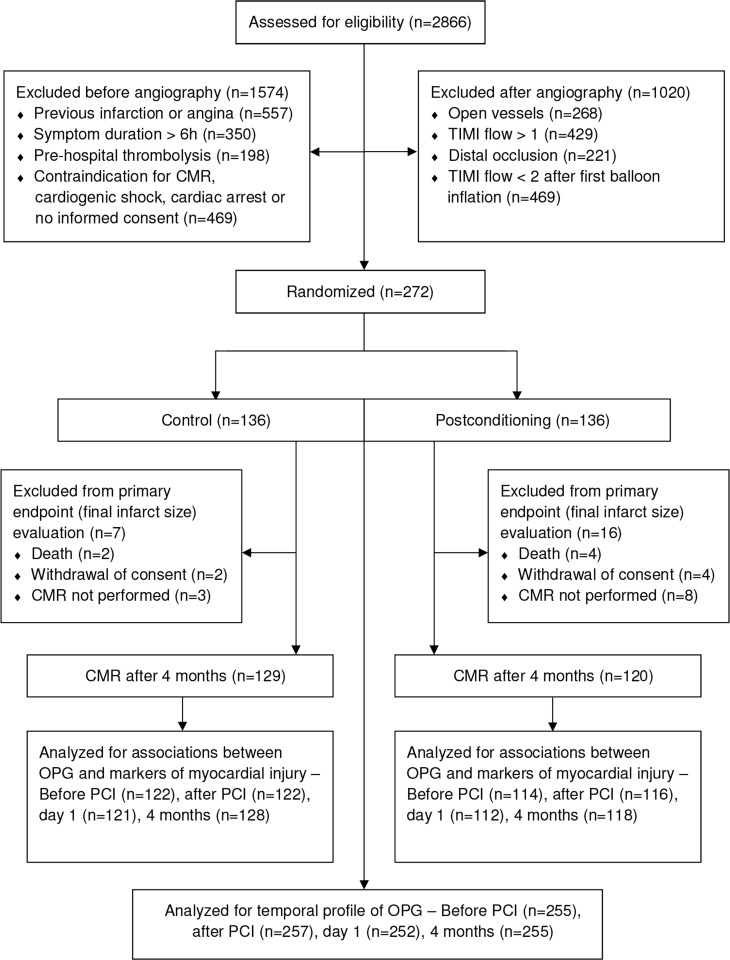
Study flow diagram.

**Fig 2:**
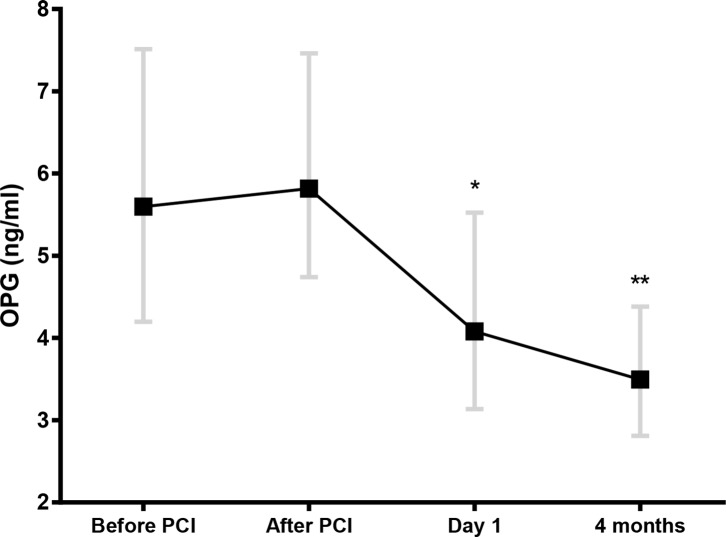
Temporal profile of osteoprotegerin (OPG) during the course of STEMI. OPG was measured in 255 patients with STEMI at the beginning and immediately after the PCI procedure, at Day 1 (median 14.7 hours after PCI), and at 4-month follow-up. Data are presented as median (boxes) with 25^th^ and 75^th^ percentile (whiskers). *p<0.001 for change in OPG levels from before PCI; **p<0.001 for both change in OPG levels from before PCI and from Day 1. **Inset:** OPG levels in patients treated with iPost or control.

Flow diagram and baseline characteristics of the study population are shown in Fig 1 and Table 1, respectively. Median age was 60 years (82% male). Whereas there was no significant change in OPG levels during PCI, there was a significant decline in OPG from before and after PCI to Day 1 and 4-month follow-up (Fig 2). Patients treated with iPost and conventional PCI had a similar temporal profile (Fig 2, inset), consequently, the study population was analyzed as a whole.

**Table 1:** Clinical and biochemical characteristics of the study population (n = 272).

Characteristics	
Age (years)	60.0 (53.0, 67.0)
Male sex	223 (82%)
Body mass index (kg/m^2^)	26.6 (24.4, 29.1)
Hypertension	73 (26.8%)
Hypercholesterolemia	26 (9.6%)
Diabetes mellitus	17 (6.3%)
Current smoker	139 (51.1%)
Time from symptom to PCI^a^ (min)	179 (123, 261)
Anterior MI^b^	131 (48.2%)
Multivessel disease	90 (33.1%)
Peak troponin T (ng/L)	5865 (3302, 10337)
Peak CRP^c^ (mg/L)	18 (7, 45)
NT-proBNP^d^ (pmol/L)	9 (5, 22)

Data are presented as median (25^th^, 75^th^ percentile) or numbers (%).

^a^PCI: Percutaneous coronary intervention

^b^Infarct localization: Anterior myocardial infarction (MI) vs inferior or posterior MI

^c^CRP: C-reactive protein

^d^NT-proBNP: N-terminal pro-B-type natriuretic peptide.

### Associations between OPG levels and LV remodeling, infarct size and LV function

We found no significant associations between OPG levels on admission (sampled before and immediately after the PCI procedure) and delta EDV, infarct size, LVEF, or myocardial salvage as determined by CMR (Table 2). OPG levels measured at Day 1 (median 14.7 hours after primary PCI), however, were significantly associated with infarct size and LVEF, both in the acute phase and at 4-month follow-up (Table 2), but not with delta EDV or myocardial salvage.

**Table 2:** Associations between osteoprotegerin (OPG) and measurements of myocardial injury and function.

	Before PCI	P-value	After PCI	P-value	Day 1	P-value	4 months	P-value
**CMR^a^ in acute phase**	n = 226		n = 229		n = 225		n = 228	
Time to CMR (days)	0.08	0.26	0.14	0.03	0.07	0.33	0.13	0.05
Infarct size (% of LV^b^ mass)	0.10	0.15	0.08	0.26	0.26	**<0.001**	0.04	0.61
Ejection fraction (%)	-0.11	0.09	-0.10	0.15	-0.30	**<0.001**	-0.08	0.26
Area at risk (% of LV)	0.05	0.47	0.10	0.15	0.25	**0.001**	0.05	0.48
Presence of MVO^c^	0.11	0.12	0.07	0.29	0.20	**0.003**	0.08	0.24
**CMR after 4 months**	n = 236		n = 238		n = 233		n = 246	
Time to CMR (months)	-0.02	0.80	-0.13	0.05	-0.12	0.08	0.01	0.92
Infarct size (% of LV mass)	0.05	0.45	0.03	0.67	0.23	**<0.001**	0.07	0.28
Ejection fraction (%)	-0.09	0.19	-0.12	0.06	-0.26	**<0.001**	-0.05	0.42
Myocardial Salvage (%)	-0.07	0.32	-0.01	0.90	-0.07	0.36	-0.04	0.59
Delta EDV^d^ (ml)	0.02	0.77	0.12	0.09	0.85	0.22	-0.03	0.66

Data are presented as Spearman correlation coefficients with p-values. Significant p-values are highlighted in bold. OPG was measured before and immediately after the PCI-procedure, at Day 1 (median 14.7 hours after PCI) and at 4-month follow-up.

^a^CMR: cardiac magnetic resonance imaging

^b^LV: left ventricle

^c^MVO: microvascular obstruction

^d^EDV: end-diastolic volume of LV.

Characteristics of the study population according to OPG levels at Day 1 are shown in Table 3. Patients with high (>median – 4.08 ng/ml) OPG levels were older, had a higher proportion of anterior MI and had higher peak TnT, peak CRP and NT-proBNP levels on admission. Patients with high OPG levels measured at Day 1 also had significantly larger infarct size, lower LVEF at 4 months, and higher frequency of MVO compared to patients with low OPG levels (Table 4). After adjustment for relevant clinical covariates in multivariable regression analyses (i.e. age, gender, time from symptom onset to PCI, infarct localization) OPG remained significantly associated with infarct size and LVEF, but not after adjustment for peak TnT and peak CRP (Table 5). The iPost procedure did not affect the association between OPG and infarct size or LVEF, respectively. OPG levels at Day 1 were significantly higher in patients with MVO compared to those without (4.29 vs 3.63 ng/ml). There were only 24 clinical events (all-cause mortality, repeat ACS, hospitalization with HF) during 4 months follow-up [19], and OPG levels at none of the sampling points were significantly different in patients with or without clinical events (data not shown).

**Table 3:** Baseline characteristics of the study population related to osteoprotegerin (OPG) levels measured at Day 1 (above or below median value).

Characteristics	OPG ≤ median	OPG > median	p-value
	n = 126	n = 126	
*Clinical characteristics*			
Age (years)	57 (49, 63)	63 (55, 70)	**<0.001**
Male sex	110 (87.3%)	100 (79.4%)	0.13
Body mass index (kg/m^2^)	27.1 (24.9, 29.3)	26.2 (24.1, 29.2)	0.16
Hypertension	30 (23.8%)	40 (31.7%)	0.21
Hypercholesterolemia	15 (11.9%)	9 (7.1%)	0.28
Diabetes mellitus	7 (5.6%)	9 (7.1%)	0.80
Current smoker	60 (47.6%)	67 (53.2%)	0.45
Time from symptom to PCI^a^(min)	185 (125, 265)	188 (126, 267)	0.69
Anterior MI^b^	52 (41.3%)	72 (57.1%)	**0.02**
Ischemic postconditioning^c^	65 (51.6%)	60 (47.6%)	0.61
*Biochemical analyses*			
Peak Troponin T (ng/L)	5326 (2965, 9037)	7027 (3834, 11831)	**0.01**
Peak CRP^d^ (mg/L)	14.7 (6.3, 32.5)	34.6 (8.6, 70.2)	**<0.01**
NT-proBNP^e^ (pmol/L)	7.0 (3.8, 16.3)	13.0 (7.0, 24.3)	**<0.001**
Creatinine (μmol/L)	70 (61, 79)	72 (63, 83)	0.24
Fasting glucose (mmol/L)	5.6 (5.3, 6.3)	6.1 (5.4, 6.8)	**<0.01**
Total cholesterol (mmol/L)	5.2 (4.6, 6.0)	5.1 (4.5, 5.9)	0.78

Data are presented as median (25^th^, 75^th^ percentile) or numbers (%). Mann-Whitney U test for continuous variables, Chi-square test for categorical variables. Significant p-values are highlighted in bold. OPG was measured median 14.7 h after the PCI-procedure.

^a^PCI: Percutaneous coronary intervention

^b^Infarct localization – Anterior myocardial infarction (MI) vs inferior or posterior MI

^c^Treated with ischemic postconditioning

^d^CRP: C-reactive protein

^e^NT-proBNP: N-terminal pro-B-type natriuretic peptide.

**Table 4:** Myocardial injury and function measured by CMR according to osteoprotegerin (OPG) values measured at Day 1.

	OPG < median	OPG > median	P-value
**CMR^a^ in acute phase**	n = 113	n = 112	
Time to CMR (days)	2 (1, 3)	2 (1, 3)	0.67
Infarct size (% of LV^b^ mass)	15.5 (9.7, 24.6)	19.7 (12.7, 33.2)	**0.004**
Ejection fraction (%)	52.0 (48.0, 59.0)	47.5 (38.5, 56.0)	**<0.001**
Area at risk (% of LV)	37.6 (31.5, 47.7)	47.1 (38.6, 55.4)	**<0.001**
Presence of MVO^c^	50 (43.9%)	60 (57.1%)	**0.05**
**CMR after 4 months**	n = 117	n = 116	
Time to CMR (months)	4 (4, 4)	4 (4, 4)	0.72
Infarct size (% of LV mass)	12.3 (6.4, 21.4)	16.1 (9.3, 24.7)	**0.01**
Ejection fraction (%)	58.0 (50.0, 63.5)	53.5 (44.5, 60.0)	**0.007**
Myocardial Salvage (%)	54.0 (40.0, 68.3)	50.4 (34.1, 67.8)	0.54
Delta EDV^d^ (ml)	9.0 (-8.3, 24.8)	9.0 (-7.0, 27.0)	0.61

Data are presented as median (25^th^, 75^th^ percentiles) or numbers (%). Mann-Whitney U test for continuous variables, Chi-square test for categorical variables. Significant p-values are highlighted in bold. OPG was measured median 14.7 hours after the PCI-procedure.

^a^CMR: cardiac magnetic resonance imaging

^b^LV: left ventricle

^c^MVO: microvascular obstruction

^d^EDV: end-diastolic volume of LV.

**Table 5:** Univariable and multivariable linear regression analyses of the associations between osteoprotegerin (OPG) measured at Day 1 and final infarct size and left ventricular ejection fraction measured by CMR at 4 months. Adjusted for clinical and biochemical covariates.

	Final infarct size			LVEF		
	*Univariable analysis*					
Variable	β	95% CI	p-value	β	95% CI	p-value
OPG^a^ (log)	15.2	6.6, 23.7	**0.001**	-18.3	-26.9, -9.7	**<0.001**
	*Multivariable analysis*					
	*Model 1*					
OPG (log)	11.9	4.1, 19.7	**<0.001**	-18.9	-27.3, -10.4	**<0.001**
Age				0.1	-0.01, 0.3	0.08
Male sex	3.6	0.1, 7.2	**0.04**	-5.7	-9.4, -2.0	**0.03**
Time from symptom to PCI^b^ (log)	10.2	3.3, 17.1	**0.004**	-9.5	-16.6, -2.3	**0.01**
Anterior MI^c^	9.6	6.9, 12.3	**<0.001**	-7.2	-10.0, -4.3	**<0.001**
	*Multivariable analysis*					
	*Model 2*					
OPG (log)	1.9	-3.7, 7.6	0.49	-9.0	-16.2, -1.9	**0.01**
Age				0.1	-0.01, 0.2	0.08
Male sex						
Time from symptom to PCI (log)						
Anterior MI	4.4	2.4, 6.4	**<0.001**	-2.9	-5.4, -0.5	**0.02**
Peak troponin T (log)	22.6	19.8, 25.4	**<0.001**	-19.3	-22.7, -15.8	**<0.001**
	*Multivariable analysis*					
	*Model 3*					
OPG (log)	0.5	-5.3, 6.3	0.87	-4.9	-12.1, -2.2	0.17
Age						
Male sex						
Time from symptom to PCI (log)						
Anterior MI	4.2	2.1, 6.2	**<0.001**	-2.6	-5.1, -0.1	**0.05**
Peak troponin T (log)	21.3	18.2, 24.4	**<0.001**	-17.7	-21.4, -13.9	**<0.001**
Peak CRP^d^ (log)	2.1	0.02, 4.1	0.06	-3.1	-5.7, -0.5	**0.02**
NT-proBNP^e^ (log)	0.01	-1.9, 1.9	0.99	0.3	-2.1, 2.6	0.83

Model 1: OPG, age, male sex, time from symptom to PCI, anterior MI. Model 2: model 1 + peak troponin t. Model 3: Model 1 + peak troponin T + peak CRP + NT-proBNP. β: unstandardized β

^a^OPG: Osteoprotegerin measured median 14.7 hours after PCI

^b^PCI: Percutaneous coronary intervention

^c^Infarct localization – Anterior myocardial infarction (MI) vs inferior or posterior MI

^d^CRP: C-reactive protein

^e^NT-proBNP: N-terminal pro-B-type natriuretic peptide. Significant p-values are highlighted in bold.

### Associations between OPG at 4-month and LV remodeling, infarct size and LV function

There were no associations between OPG levels at 4 months and any measurement of LV injury or remodeling measured by CMR at the same follow-up (Table 2).

### Heparin effect on OPG levels

**Fig 3:**
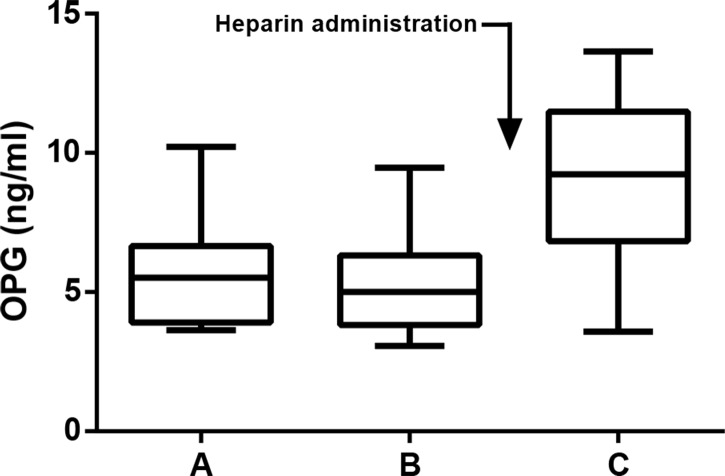
Osteoprotegerin (OPG) levels in patients with stable coronary artery disease (CAD) before and after heparin administration. Blood samples were drawn from patients (n = 20) during elective coronary angiography. A venous sample was taken before angiography (**A**, median 72 min before heparin). Arterial samples were taken immediately after cannulation (**B**, 1 min before heparin), and at the end of the angiography procedure (**C**, 20 min after heparin). Data are presented as boxplots, with median (line), 25^th^ and 75^th^ percentile (box) and range (whiskers).

Based on the lack of association between OPG levels very early after admission and measurements of ischemic injury by CMR, a possible relation between OPG levels and heparin administration was studied in 20 patients with stable CAD. There was a substantial increase in OPG levels following heparin administration with a median increase of 77% (p<0.0001, Fig 3). There was no difference between OPG levels measured in the venous samples compared to the arterial samples before heparin administration. There was no significant difference in OPG levels between patients treated with PCI (n = 5) compared to patients with coronary angiography only (n = 15) (data not shown).

## Discussion

The main finding of the study was that OPG measured in STEMI patients was not associated with LV remodeling or myocardial salvage. However, high levels of OPG measured the first day after PCI were associated with larger final infarct size and lower LVEF 4 months after STEMI, also after adjustment for several clinical variables, but not after adjustment for peak TnT and CRP. A temporal profile demonstrated high levels at admission and immediately after PCI, but these levels were not related to myocardial injury or function measured by CMR 4 months later. This might be related to confounding effects of heparin administration.

The role of OPG in STEMI is largely unknown. Rapid and sustained release of OPG from vascular endothelial cells has been demonstrated in response to inflammatory stimuli, suggesting a modulatory role of OPG in vascular injury, inflammation and hemostasis [26]. STEMI is known to launch a massive inflammatory response, and it is possible that this could contribute to the increased OPG levels seen in STEMI patients. Persistent and excessive inflammation independent of infarct size has been suggested to contribute to adverse LV remodeling following MI [27]. In an experimental model of post-infarction HF, increased myocardial expression of OPG has been reported, with increased gene expression in both the ischemic and non-ischemic part of the LV [28], suggesting a role of OPG in maladaptive remodeling following MI. Moreover, OPG has been found to be a predictor of adverse outcomes in ACS including development of HF [11]. In the present study we found no association between OPG levels and adverse remodeling measured as delta EDV.

Our findings partially support studies reporting an association between OPG levels and the extent of myocardial injury in STEMI patients [14, 15, 17]. Thus, patients with high OPG levels at Day 1 had larger area at risk, final infarct size, lower LVEF, and a higher frequency of MVO. The associations between OPG and infarct size and LVEF at 4 months in our study, however, were not present after adjustment for peak TnT and CRP, which reflect myocardial necrosis and inflammation, respectively. It is possible that levels of OPG mainly reflect these processes. Moreover, the fact that we used peak values of troponin T and CRP in the multivariable analyses may also have contributed to the superior prognostic information of these biomarkers in the present study.

Some investigators have suggested that a chronic persistent inflammatory state may reflect adverse LV remodeling and that biomarkers measured late after MI may reflect mechanisms other than acute necrosis [27]. OPG measured in stable STEMI patients 4 months after the index infarction, however, was not associated with indices of LV remodeling such as change in EDV or LVEF in the present study. The number of patients with clinical events during 4 months follow-up was low and could not be related to OPG levels [19].

Contrary to previous reports [15, 17, 29], OPG levels measured before and shortly after the PCI procedure were not related to myocardial injury or impaired function, possibly masked by heparin related release of OPG. OPG levels were significantly higher during the PCI procedure compared to levels on Day 1 after admission, in line with previous reports [17]. OPG contains a heparin-binding domain [30], and it has been proposed that the high levels of OPG in the initial phase of STEMI are related to heparin administration. *In vitro* studies have indeed demonstrated rapid release of OPG from smooth muscle cells after heparin treatment [31]. Moreover, in a small study in healthy individuals, the investigators reported a 2-fold increase in OPG levels within 5 minutes following intravenous heparin infusion, normalizing within 1 hour after the infusion [32]. In all of the patients in the POSTEMI study, heparin was administered in the prehospital setting or in the cath. lab prior to the initial blood sampling. Our results suggest that heparin administration has a major influence on OPG levels in patients with stable CAD with no additional effect of PCI. It is therefore likely that the high OPG levels measured early in STEMI patients reflect the heparin effect, and not the ischemic injury only and this should clearly be taken into account when evaluating OPG levels in MI patients early after admission.

### Limitations

Associations between OPG and CMR measurements of myocardial injury and function were lost after adjustment for known prognostic indicators such as TnT and CRP, suggesting a limited role of OPG in risk stratification in STEMI patients. The number of included patients was relatively low. In particular, due to the relatively low-risk population and few clinical endpoints, the study was not powered to elucidate a possible association between OPG and clinical events. Due to the explorative nature of the study, no correction for multiple comparisons was performed and this could possibly limit the conclusions drawn from the results. Moreover, although the heparin effect on OPG levels has been reported to last for only a few hours [32], the lack of data on the duration of the heparin effect is a weakness with this part of the study. It was not feasible to have a control group in the heparin study due to safety concerns. Consequently, we cannot rule out that other aspects of the PCI procedure than the heparin administration contributed to the OPG release. However, the lack of increase in OPG levels after PCI in the POSTEMI patients (all received heparin before the first sample) indicates that this is not the most likely explanation.

## Conclusions

Our findings indicate that high levels of OPG are associated with myocardial injury, but not adverse remodeling or myocardial salvage. The role of OPG as a potential biomarker in STEMI patients to identify patients with risk of adverse remodeling and HF development seems to be limited by a strong association with age, confounding effect of heparin administration, and little additive value to well-established biomarkers such as Troponin T, NT-pro-BNP and CRP.

## Supporting information

S1 Checklist Strobe checklist. (PDF)

S1 File Design article. (PDF)

S2 File Supporting data set. (XLSX)

S1 Protocol POSTEMI study protocol. (PDF)

## References

[1] Hofbauer LC, Khosla S, Dunstan CR, Lacey DL, Boyle WJ, Riggs BL. The roles of osteoprotegerin and osteoprotegerin ligand in the paracrine regulation of bone resorption. Journal of bone and mineral research: the official journal of the American Society for Bone and Mineral Research. 2000;15(1):2–12. 10.1359/jbmr.2000.15.1.2 10646108

[2] Emery JG, McDonnell P, Burke MB, Deen KC, Lyn S, Silverman C, et al Osteoprotegerin is a receptor for the cytotoxic ligand TRAIL. The Journal of biological chemistry. 1998;273(23):14363–7. 9603945 10.1074/jbc.273.23.14363

[3] Collin-Osdoby P. Regulation of vascular calcification by osteoclast regulatory factors RANKL and osteoprotegerin. Circ Res. 2004;95(11):1046–57. 10.1161/01.RES.0000149165.99974.12 15564564

[4] Schoppet M, Preissner KT, Hofbauer LC. RANK ligand and osteoprotegerin: paracrine regulators of bone metabolism and vascular function. Arterioscler Thromb Vasc Biol. 2002;22(4):549–53. 11950689 10.1161/01.atv.0000012303.37971.da

[5] Kiechl S, Schett G, Wenning G, Redlich K, Oberhollenzer M, Mayr A, et al Osteoprotegerin is a risk factor for progressive atherosclerosis and cardiovascular disease. Circulation. 2004;109(18):2175–80. 10.1161/01.CIR.0000127957.43874.BB 15117849

[6] Vik A, Mathiesen EB, Brox J, Wilsgaard T, Njolstad I, Jorgensen L, et al Serum osteoprotegerin is a predictor for incident cardiovascular disease and mortality in a general population: the Tromso Study. J Thromb Haemost. 2011;9(4):638–44. 10.1111/j.1538-7836.2011.04222.x 21284802

[7] Abedin M, Omland T, Ueland T, Khera A, Aukrust P, Murphy SA, et al Relation of osteoprotegerin to coronary calcium and aortic plaque (from the Dallas Heart Study). Am J Cardiol. 2007;99(4):513–8. 10.1016/j.amjcard.2006.08.064 17293196

[8] Jono S, Ikari Y, Shioi A, Mori K, Miki T, Hara K, et al Serum osteoprotegerin levels are associated with the presence and severity of coronary artery disease. Circulation. 2002;106(10):1192–4. 12208791 10.1161/01.cir.0000031524.49139.29

[9] Schoppet M, Sattler AM, Schaefer JR, Herzum M, Maisch B, Hofbauer LC. Increased osteoprotegerin serum levels in men with coronary artery disease. J Clin Endocrinol Metab. 2003;88(3):1024–8. 10.1210/jc.2002-020775 12629080

[10] Sandberg WJ, Yndestad A, Oie E, Smith C, Ueland T, Ovchinnikova O, et al Enhanced T-cell expression of RANK ligand in acute coronary syndrome: possible role in plaque destabilization. Arterioscler Thromb Vasc Biol. 2006;26(4):857–63. 10.1161/01.ATV.0000204334.48195.6a 16424351

[11] Omland T, Ueland T, Jansson AM, Persson A, Karlsson T, Smith C, et al Circulating osteoprotegerin levels and long-term prognosis in patients with acute coronary syndromes. J Am Coll Cardiol. 2008;51(6):627–33. 10.1016/j.jacc.2007.09.058 18261681

[12] Ueland T, Jemtland R, Godang K, Kjekshus J, Hognestad A, Omland T, et al Prognostic value of osteoprotegerin in heart failure after acute myocardial infarction. J Am Coll Cardiol. 2004;44(10):1970–6. 10.1016/j.jacc.2004.06.076 15542278

[13] Crisafulli A, Micari A, Altavilla D, Saporito F, Sardella A, Passaniti M, et al Serum levels of osteoprotegerin and RANKL in patients with ST elevation acute myocardial infarction. Clin Sci (Lond). 2005;109(4):389–95. 15926884 10.1042/CS20050058

[14] Andersen GO, Knudsen EC, Aukrust P, Yndestad A, Oie E, Muller C, et al Elevated serum osteoprotegerin levels measured early after acute ST-elevation myocardial infarction predict final infarct size. Heart. 2011;97(6):460–5. 10.1136/hrt.2010.206714 21270073

[15] Fuernau G, Zaehringer S, Eitel I, de Waha S, Droppa M, Desch S, et al Osteoprotegerin in ST-elevation myocardial infarction: Prognostic impact and association with markers of myocardial damage by magnetic resonance imaging. International Journal of Cardiology. 2013;167(5):2134–9. 10.1016/j.ijcard.2012.05.101 22704876

[16] Bjerre M, Munk K, Sloth AD, Nielsen SS, Flyvbjerg A, Botker HE. High osteoprotegerin levels predict MACCE in STEMI patients, but are not associated with myocardial salvage. Scand Cardiovasc J. 2014;48(4):209–15. Epub 2014/04/25. 10.3109/14017431.2014.917767 24758546

[17] Lindberg S, Jensen JS, Hoffmann S, Iversen AZ, Pedersen SH, Mogelvang R, et al Osteoprotegerin levels change during STEMI and reflect cardiac function. Can J Cardiol. 2014;30(12):1523–8. 10.1016/j.cjca.2014.08.015 25475457

[18] Limalanathan S, Andersen GO, Hoffmann P, Klow NE, Abdelnoor M, Eritsland J. Rationale and design of the POSTEMI (postconditioning in ST-elevation myocardial infarction) study. Cardiology. 2010;116(2):103–9. 10.1159/000316965 20588018

[19] Limalanathan S, Andersen GO, Klow NE, Abdelnoor M, Hoffmann P, Eritsland J. Effect of ischemic postconditioning on infarct size in patients with ST-elevation myocardial infarction treated by primary PCI results of the POSTEMI (POstconditioning in ST-Elevation Myocardial Infarction) randomized trial. J Am Heart Assoc. 2014;3(2):e000679 PubMed Central PMCID: PMC4187468. 10.1161/JAHA.113.000679 24760962 PMC4187468

[20] Task Force on the management of STseamiotESoC, Steg PG, James SK, Atar D, Badano LP, Blomstrom-Lundqvist C, et al ESC Guidelines for the management of acute myocardial infarction in patients presenting with ST-segment elevation. Eur Heart J. 2012;33(20):2569–619. 10.1093/eurheartj/ehs215 22922416

[21] Ueland T, Bollerslev J, Godang K, Muller F, Froland SS, Aukrust P. Increased serum osteoprotegerin in disorders characterized by persistent immune activation or glucocorticoid excess—possible role in bone homeostasis. Eur J Endocrinol. 2001;145(6):685–90. 11720891 10.1530/eje.0.1450685

[22] Limalanathan S, Eritsland J, Andersen GO, Klow NE, Abdelnoor M, Hoffmann P. Myocardial salvage is reduced in primary PCI-treated STEMI patients with microvascular obstruction, demonstrated by early and late CMR. PLoS One. 2013;8(8):e71780 PubMed Central PMCID: PMC3747268. 10.1371/journal.pone.0071780 23977143 PMC3747268

[23] Eitel I, Desch S, Fuernau G, Hildebrand L, Gutberlet M, Schuler G, et al Prognostic significance and determinants of myocardial salvage assessed by cardiovascular magnetic resonance in acute reperfused myocardial infarction. J Am Coll Cardiol. 2010;55(22):2470–9. 10.1016/j.jacc.2010.01.049 20510214

[24] Desch S, Engelhardt H, Meissner J, Eitel I, Sareban M, Fuernau G, et al Reliability of myocardial salvage assessment by cardiac magnetic resonance imaging in acute reperfused myocardial infarction. Int J Cardiovasc Imaging. 2012;28(2):263–72. 10.1007/s10554-011-9802-9 21279689

[25] Masci PG, Ganame J, Strata E, Desmet W, Aquaro GD, Dymarkowski S, et al Myocardial salvage by CMR correlates with LV remodeling and early ST-segment resolution in acute myocardial infarction. JACC Cardiovasc Imaging. 2010;3(1):45–51. 10.1016/j.jcmg.2009.06.016 20129530

[26] Zannettino AC, Holding CA, Diamond P, Atkins GJ, Kostakis P, Farrugia A, et al Osteoprotegerin (OPG) is localized to the Weibel-Palade bodies of human vascular endothelial cells and is physically associated with von Willebrand factor. J Cell Physiol. 2005;204(2):714–23. 10.1002/jcp.20354 15799029

[27] Westman PC, Lipinski MJ, Luger D, Waksman R, Bonow RO, Wu E, et al Inflammation as a Driver of Adverse Left Ventricular Remodeling After Acute Myocardial Infarction. Journal of the American College of Cardiology. 2016;67(17):2050–60. 10.1016/j.jacc.2016.01.073 27126533

[28] Ueland T, Yndestad A, Oie E, Florholmen G, Halvorsen B, Froland SS, et al Dysregulated osteoprotegerin/RANK ligand/RANK axis in clinical and experimental heart failure. Circulation. 2005;111(19):2461–8. 10.1161/01.CIR.0000165119.62099.14 15883214

[29] Erkol A, Oduncu V, Pala S, Kizilirmak F, Kilicgedik A, Yilmaz F, et al Plasma osteoprotegerin level on admission is associated with no-reflow phenomenon after primary angioplasty and subsequent left ventricular remodeling in patients with acute ST-segment elevation myocardial infarction. Atherosclerosis. 2012;221(1):254–9. 10.1016/j.atherosclerosis.2011.12.031 22265272

[30] Yamaguchi K, Kinosaki M, Goto M, Kobayashi F, Tsuda E, Morinaga T, et al Characterization of structural domains of human osteoclastogenesis inhibitory factor. J Biol Chem. 1998;273(9):5117–23. 9478964 10.1074/jbc.273.9.5117

[31] Nybo M, Rasmussen LM. Osteoprotegerin released from the vascular wall by heparin mainly derives from vascular smooth muscle cells. Atherosclerosis. 2008;201(1):33–5. 10.1016/j.atherosclerosis.2008.03.026 18490020

[32] Vik A, Brodin E, Sveinbjornsson B, Hansen JB. Heparin induces mobilization of osteoprotegerin into the circulation. Thromb Haemost. 2007;98(1):148–54. 17598007

