# Supplementary material for: Osteoprotegerin levels in ST-elevation myocardial infarction: Temporal profile and association with myocardial injury and left ventricular function
Source: PLoS One. 2017 Mar 2;12(3):e0173034. doi: 10.1371/journal.pone.0173034 (PMC5333871; doi:10.1371/journal.pone.0173034)
Supplement: S1 Checklist — (PDF) [file pone.0173034.s001.pdf]

STROBE Statement—checklist of items that should be included in reports of observational studies

	Item No.	Recommendation	Page No.	Relevant text from manuscript
**Title and abstract**	1	(*a*) Indicate the study's design with a commonly used term in the title or the abstract	2	**Abstract**: "The aims of this **observational cohort study** were to evaluate: 1) the temporal profile of OPG during STEMI, 2) possible associations between OPG measured acutely and after 4 months, with infarct size, adverse left ventricular (LV) remodeling, microvascular obstruction (MVO) and myocardial salvage and 3) the effect of heparin administration on OPG levels."
		(*b*) Provide in the abstract an informative and balanced summary of what was done and what was found	2	**Abstract, Methods and results: "**Blood samples were drawn repeatedly from 272 STEMI patients treated with primary percutaneous coronary intervention (PCI). Cardiac magnetic resonance imaging (CMR) was performed in the acute phase and after 4 months. The effect of heparin administration on OPG levels was studied in 20 patients referred to elective coronary angiography.OPG levels measured acutely were significantly higher than Day 1 and during follow-up. OPG levels were correlated with age. No association was found between early OPG levels and CMR measurements at 4 months. Patients with >median OPG levels measured at Day 1 had larger final infarct size, lower LV ejection fraction (LVEF) at 4 months and higher frequency of MVO. There were no associations between OPG and change in end-diastolic volume or myocardial salvage. OPG remained associated with infarct size and LVEF after adjustment for relevant covariates, except peak troponin T and CRP. A 77% increase in OPG levels following heparin administration was found in patients undergoing elective coronary angiography."
**Introduction**				
Background/rationale	2	Explain the scientific background and rationale for the investigation being reported	4	**Introduction, opening paragraphs**: "Osteoprotegerin (OPG), a glycoprotein in the tumor necrosis factor (TNF) receptor superfamily, acts as a decoy receptor for the receptor activator of nuclear factor-κB ligand (RANKL) and TNF-related apoptosis inducing ligand (TRAIL) (1, 2). OPG is expressed in most human tissues and cells, including bone (osteoblasts), vascular smooth muscle cells, and endothelial cells (3, 4). Increased levels of OPG have been associated with coronary calcium score and the development and severity of coronary artery disease (CAD) (5-9). Moreover, OPG, as well as RANKL, are expressed within the failing myocardium in both experimental and clinical

				heart failure (HF), and strong immunostaining of these molecules has also been found within atherosclerotic carotid plaques as well as in thrombus material obtained at the site of plaque rupture during myocardial infarction (MI) (10). In addition, OPG has been identified as an independent predictor of HF development and mortality in patients with acute coronary syndrome (ACS) (11, 12). Elevated serum OPG has been reported in ST-elevation MI (STEMI) patients compared to patients with non-STEMI, unstable angina, stable CAD, and controls (11, 13). We have previously shown, in a study of 199 STEMI patients, that OPG levels measured a median of 16.5 hours after percutaneous coronary intervention (PCI) were significantly associated with infarct size assessed after 3 months by SPECT imaging (14). However, the results of more recent studies investigating OPG and infarct size in STEMI patients have been inconsistent (15-17)."
Objectives	3	State specific objectives, including any prespecified hypotheses	4-5	**Introduction, following paragraphs**: "The objectives of the present study were therefore: 1) to establish a temporal profile of OPG in STEMI, 2) to examine the association of OPG with markers of adverse left ventricular (LV) remodeling in patients with STEMI, 3) to investigate possible associations of OPG measured during acute STEMI with final infarct size, microvascular obstruction (MVO) and myocardial salvage, 4) to study a possible association between OPG levels measured in a clinically stable situation late after STEMI and adverse LV remodeling, and 5) to elucidate the effect of heparin administration on OPG levels."
**Methods**				
Study design	4	Present key elements of study design early in the paper	5-6	Full study protocol provided in the supporting information. **Methods, study population:** "The POSTEMI trial was a prospective, randomized, single-center, open-label clinical trial investigating the cardioprotective strategy ischemic postconditioning (iPost)."
Setting	5	Describe the setting, locations, and relevant dates, including periods of recruitment, exposure, follow-up, and data collection	6	**Material and Methods, study population:** "Briefly, the study population consisted of 272 patients with first-time STEMI and symptom duration < 6 hours included between January 12, 2009, and August 25, 2012, at Oslo University Hospital Ullevål,"
Participants	6	(*a*) *Cohort study*—Give the eligibility criteria, and the sources and methods of selection of participants. Describe methods of follow-up	6	**Material and Methods, study population:** "Patients with inability to provide informed consent, previous MI, renal failure (serum creatinine >200 µmol/L), contraindications for cardiac magnetic resonance imaging (CMR), and

		Case-control study—Give the eligibility criteria, and the sources and methods of case ascertainment and control selection. Give the rationale for the choice of cases and controls<br>*Cross-sectional study*—Give the eligibility criteria, and the sources and methods of selection of participants		clinically unstable patients (cardiac arrest, cardiogenic shock, pulmonary congestion, or hypotension) were excluded. Occlusion (Thrombolysis In Myocardial Infarction [TIMI] flow 0-1) of the proximal/middle part of one of the three main coronary vessels with no/minimal collateral flow to the ischemic myocardium and successful reperfusion after the first balloon inflation (TIMI flow 2-3) had to be demonstrated angiographically before 1:1 randomization to iPost or control group."
		(*b*) *Cohort study*—For matched studies, give matching criteria and number of exposed and unexposed<br>*Case-control study*—For matched studies, give matching criteria and the number of controls per case		Not applicable
Variables	7	Clearly define all outcomes, exposures, predictors, potential confounders, and effect modifiers. Give diagnostic criteria, if applicable	7	**Material and Methods, CMR protocol and analysis**: Definition of outcome variables.
Data sources/ measurement	8*	For each variable of interest, give sources of data and details of methods of assessment (measurement). Describe comparability of assessment methods if there is more than one group	7-8	**Biochemical analysis:** Description of lab methods. "Serum levels of OPG were quantified by enzyme immunoassay using matched antibodies from R&D Systems (Minneapolis, Minnesota, USA) as previously described and validated (21). The intra-assay and inter-assay coefficients of variation (CV) were <10%. The sensitivity was calculated to be 15 pg/ml."<br>**Material and Methods, CMR protocol and analysis**: Description of the imaging methods. "The details regarding CMR protocol and analyses have been published in detail previously (22). In brief, a 1.5 T scanner (Philips Intera, release 11 or Philips Achieva, release 3.2, Best, Netherlands) was used to obtain images, and image analyses were performed on an extended MR Work Space (Philips Medical Systems). Short axis images of LV were acquired for complete volume analysis including LV ejection fraction (LVEF). T2 weighted imaging was performed in the short axis plane to quantify the area at risk, defined as myocardium with a signal intensity (SI) of more than 2 standard deviations above the SI in remote non-infarcted myocardium. Late gadolinium enhancement (LGE) imaging was obtained 15 minutes after contrast injection (Gadolinium-DTPA 469 mg/ml, 0.15 mmol/kg, Magnevist, Schering AG, Germany), and two and four chamber long axis views and short axis views were assessed to determine infarct size.

				Myocardial salvage index (%) was calculated as follows: [(area at risk – infarct size at 4 months)/area at risk] x 100 (23-25). MVO was assessed in late enhancement images, and defined as a dark area within the hyperintense area in the infarcted myocardium."<br>**Relevant data set provided in the supporting information.**
Bias	9	Describe any efforts to address potential sources of bias	6-8	**Material and Methods, Study population:** Study design: "The POSTEMI trial was a prospective, randomized, single-center, open-label clinical trial investigating the cardioprotective strategy ischemic postconditioning (iPost). The study design has previously been reported in detail (18, 19)."<br>**Material and Methods, Biochemical analyses:** Measurement errors: "Serum levels of OPG were quantified by enzyme immunoassay using matched antibodies from R&D Systems (Minneapolis, Minnesota, USA) as previously described and validated (21). The intra-assay and inter-assay coefficients of variation (CV) were <10%. The sensitivity was calculated to be 15 pg/ml. C-reactive protein (CRP) was determined by routine laboratory high-sensitivity assay, peak CRP was defined as the maximum value measured during hospitalization. Levels of N-terminal pro-B-type natriuretic peptide (NT-proBNP) on admission were determined with Elecsys proBNP sandwich immunoassay on Elecsys 2010 (Roche Diagnostics). Serum cardiac-specific troponin T (TnT) was measured by electrochemiluminescence technology for quantitative measurement (Elecsys 2010, Roche, Mannheim, Germany). The peak TnT level was defined as the maximum value measured during hospitalization. Inter-assay and intra-assay CV% were <7 % for all assays."<br>**Material and Methods, Statistical analyses:** Possible confounding: "The following covariates were entered into the models based on either clinical relevance or an association with either OPG or the dependent variable with a p-value < 0.2: Age, gender, time from symptom onset to PCI, infarct localization (anterior MI vs inferior or posterior MI), treatment with ischemic postconditioning, peak TnT, peak CRP and NT-proBNP on admission."
Study size	10	Explain how the study size was arrived at		Not applicable: This was an observational study of a set cohort.

Continued on next page

Quantitative variables	11	Explain how quantitative variables were handled in the analyses. If applicable, describe which groupings were chosen and why	8	**Material and Methods, Statistical analyses, opening paragraphs:** "OPG was analyzed both as a continuous variable and as a dichotomized variable. Analyses of the association between OPG dichotomized at each quartile and end-diastolic volume (EDV), infarct size, LVEF, and myocardial salvage index resulted in different cut-off values for the different outcome variables. The median value was chosen as cut-off for all outcome variables due to considerations of power, and to make interpretation of the results easier."
Statistical methods	12	(*a*) Describe all statistical methods, including those used to control for confounding	8	**Material and Methods, Statistical analyses, following paragraphs:** "Non-parametric tests were used for group comparisons, due to skewness in some of the analyzed variables. Mann Whitney U test was used for group analyses of continuous variables, while categorical variables were analyzed using Chi-square test. Wilcoxon signed rank test was used to compare OPG levels at different sampling points. Multivariable linear regression analyses were performed with final infarct size, LVEF, myocardial salvage index, and delta EDV (change from baseline to 4-month) as outcome variables, respectively."
		(*b*) Describe any methods used to examine subgroups and interactions		Not applicable
		(*c*) Explain how missing data were addressed	8	**Material and Methods, Statistical analyses, following paragraphs:** "Pairwise deletion was used to handle missing data in multivariable analyses."
		(*d*) *Cohort study*—If applicable, explain how loss to follow-up was addressed<br>*Case-control study*—If applicable, explain how matching of cases and controls was addressed<br>*Cross-sectional study*—If applicable, describe analytical methods taking account of sampling strategy		Not applicable
		(*e*) Describe any sensitivity analyses		A sensitivity analysis was performed to assess the impact of the chosen missing data procedure. We re-did the regression analyses using listwise deletion with similar results as with pairwise deletion.
**Results**				
Participants	13*	(a) Report numbers of individuals at each stage of study—eg numbers potentially eligible, examined for	9	**Results, first paragraph**: Provided in flow diagram of the study in Figure 1.

		eligibility, confirmed eligible, included in the study, completing follow-up, and analysed		
		(b) Give reasons for non-participation at each stage	9	Addressed in Figure 1.
		(c) Consider use of a flow diagram	9	See 13(a) and (b)
Descriptive data	14*	(a) Give characteristics of study participants (eg demographic, clinical, social) and information on exposures and potential confounders	9	Baseline characteristics provided in Table 1.
		(b) Indicate number of participants with missing data for each variable of interest		Participants with missing data for the baseline characteristics were negligible and thus we did not report these values in the manuscript.
		(c) *Cohort study*—Summarise follow-up time (eg, average and total amount)		Not applicable
Outcome data	15*	*Cohort study*—Report numbers of outcome events or summary measures over time	11-14	**Results**, *Associations between OPG levels and LV remodeling, infarct size and LV function:* CMR outcomes provided in Table 4. **Results**, *Associations between OPG levels and LV remodeling, infarct size and LV function*: Clinical events reported: "There were only 24 clinical events (all-cause mortality, repeat ACS, hospitalization with HF) during 4 months follow-up (19)".
		Case-control study—Report numbers in each exposure category, or summary measures of exposure		Not applicable
		Cross-sectional study—Report numbers of outcome events or summary measures		Not applicable
Main results	16	(*a*) Give unadjusted estimates and, if applicable, confounder-adjusted estimates and their precision (eg, 95% confidence interval). Make clear which confounders were adjusted for and why they were included	13-14	**Results:** Univariable and multivariable regression analyses provided in Table 5.
		(*b*) Report category boundaries when continuous variables were categorized		See 11 above
		(*c*) If relevant, consider translating estimates of relative risk into absolute risk for a meaningful time period		Not applicable

Continued on next page

Other analyses	17	Report other analyses done—eg analyses of subgroups and interactions, and sensitivity analyses		Not applicable
**Discussion**				
Key results	18	Summarise key results with reference to study objectives	9<br><br><br><br><br>10-14<br><br><br><br><br><br><br><br><br><br><br><br><br><br><br><br><br><br><br><br><br>15	**Results**, *Temporal profile of OPG*: "Whereas there was no significant change in OPG levels during PCI, there was a significant decline in OPG from before and after PCI to Day 1 and 4-month follow-up (Fig 2). Patients treated with iPost and conventional PCI had a similar temporal profile (Fig 2, inset)."<br>**Results**, *Associations between OPG levels and LV remodeling, infarct size and LV function*: "We found no significant associations between OPG levels on admission (sampled before and immediately after the PCI procedure) and delta EDV, infarct size, LVEF, or myocardial salvage as determined by CMR (Table 2). OPG levels measured at Day 1 (median 14.7 hours after primary PCI), however, were significantly associated with infarct size and LVEF, both in the acute phase and at 4-month follow-up (Table 2), but not with delta EDV or myocardial salvage. Characteristics of the study population according to OPG levels at Day 1 are shown in Table 3. Patients with high (>median – 4.08 ng/ml) OPG levels were older, had a higher proportion of anterior MI and had higher peak TnT, peak CRP and NT-proBNP levels on admission. Patients with high OPG levels measured at Day 1 also had significantly larger infarct size, lower LVEF at 4 months, and higher frequency of MVO compared to patients with low OPG levels (Table 4). After adjustment for relevant clinical covariates in multivariable regression analyses (i.e. age, gender, time from symptom onset to PCI, infarct localization) OPG remained significantly associated with infarct size and LVEF, but not after adjustment for peak TnT and peak CRP (Table 5). The iPost procedure did not affect the association between OPG and infarct size or LVEF, respectively. OPG levels at Day 1 were significantly higher in patients with MVO compared to those without (4.29 vs 3.63 ng/ml)." Additional results provided in Table 4 and 5.<br>**Results,** *Heparin effect on OPG levels*: "Based on the lack of association between OPG levels very early after admission and measurements of ischemic injury by CMR, a possible relation between OPG levels and heparin administration was studied in 20 patients with stable CAD. There was a substantial increase in OPG levels following heparin administration

				with a median increase of 77% (p<0.0001, Fig 3). There was no difference between OPG levels measured in the venous samples compared to the arterial samples before heparin administration. There was no significant difference in OPG levels between patients treated with PCI (n=5) compared to patients with coronary angiography only (n=15) (data not shown)."
Limitations	19	Discuss limitations of the study, taking into account sources of potential bias or imprecision. Discuss both direction and magnitude of any potential bias	17-18	**Discussion**, *Limitations*: "Associations between OPG and CMR measurements of myocardial injury and function were lost after adjustment for known prognostic indicators such as TnT and CRP, suggesting a limited role of OPG in risk stratification in STEMI patients. The number of included patients was relatively low. In particular, due to the relatively low-risk population and few clinical endpoints the study was not powered to elucidate a possible association between OPG and clinical events. Due to the explorative nature of the study, no correction for multiple comparisons was performed and this could possibly limit the conclusions drawn from the results. Moreover, although the heparin effect on OPG levels has been reported to last for only a few hours (32), the lack of data on the duration of the heparin effect is a weakness with this part of the study. It was not feasible to have a control group in the heparin study due to safety concerns. Consequently, we cannot rule out that other aspects of the PCI procedure than the heparin administration contributed to the OPG release. However, the lack of increase in OPG levels after PCI in the POSTEMI patients (all had received heparin before the first sample) indicates that this is not the most likely explanation."
Interpretation	20	Give a cautious overall interpretation of results considering objectives, limitations, multiplicity of analyses, results from similar studies, and other relevant evidence	16-17	**Discussion with limitations:** "In an experimental model of post-infarction HF, increased myocardial expression of OPG has been reported, with increased gene expression in both the ischemic and non-ischemic part of the LV (28), suggesting a role of OPG in maladaptive remodeling following MI. Moreover, OPG has been found to be a predictor of adverse outcomes in ACS including development of HF (11). In the present study we found no association between OPG levels and adverse remodeling measured as delta EDV. Our findings partially support studies reporting an association between OPG levels and the extent of myocardial injury in STEMI patients (14, 15, 17). Thus, patients with high OPG levels at Day 1 had larger area at risk, final infarct size, lower LVEF, and a higher frequency of MVO. The associations between OPG and infarct size and LVEF at 4 months in our study, however, were not present after adjustment for peak TnT and CRP,

which reflect myocardial necrosis and inflammation, respectively. It is possible that levels of OPG mainly reflect these processes. Moreover, the fact that we used peak values of troponin T and CRP in the multivariable analyses may also have contributed to the superior prognostic information of these biomarkers in the present study.Some investigators have suggested that a chronic persistent inflammatory state may reflect adverse LV remodeling and that biomarkers measured late after MI may reflect mechanisms other than acute necrosis (27). OPG measured in stable STEMI patients 4 months after the index infarction, however, was not associated with indices of LV remodeling such as change in EDV or LVEF in the present study. The number of patients with clinical events during 4 months follow-up was low and could not be related to OPG levels (19). Contrary to previous reports (15, 17, 29), OPG levels measured before and shortly after the PCI procedure were not related to myocardial injury or impaired function, possibly masked by heparin related release of OPG. OPG levels were significantly higher during the PCI procedure compared to levels on Day 1 after admission, in line with previous reports (17). OPG contains a heparin-binding domain (30), and it has been proposed that the high levels of OPG in the initial phase of STEMI are related to heparin administration. *In vitro* studies have indeed demonstrated rapid release of OPG from smooth muscle cells after heparin treatment (31). Moreover, in a small study in healthy individuals, the investigators reported a 2-fold increase in OPG levels within 5 minutes following intravenous heparin infusion, normalizing within 1 hour after the infusion (32). In all of the patients in the POSTEMI study, heparin was administered in the prehospital setting or in the cath. lab prior to the initial blood sampling. Our results suggest that heparin administration has a major influence on OPG levels in patients with stable CAD with no additional effect of PCI. It is therefore likely that the high OPG levels measured early in STEMI patients reflect the heparin effect, and not the ischemic injury only and this should clearly be taken into account when evaluating OPG levels in MI patients early after admission.

18 **Conclusions**: “Our findings indicate that high levels of OPG are associated with myocardial injury, but not adverse remodeling or myocardial salvage. The role of OPG as a potential biomarker in STEMI patients to identify patients with risk of adverse remodeling and HF development seems to be limited by a strong association with age, confounding effect of heparin

				administration, and little additive value to well-established biomarkers such as Troponin T, NT-pro-BNP and CRP.”
Generalisability	21	Discuss the generalisability (external validity) of the study results	16-18	Addressed in the Discussion and Limitations sections.
**Other information**				
Funding	22	Give the source of funding and the role of the funders for the present study and, if applicable, for the original study on which the present article is based		**Sources of Funding**: “This work was supported by The Norwegian Health and Rehabilitation Foundation and Center for Heart Failure Research, University of Oslo, Oslo, Norway.”

*Give information separately for cases and controls in case-control studies and, if applicable, for exposed and unexposed groups in cohort and cross-sectional studies.

Note: An Explanation and Elaboration article discusses each checklist item and gives methodological background and published examples of transparent reporting. The STROBE checklist is best used in conjunction with this article (freely available on the Web sites of PLoS Medicine at http://www.plosmedicine.org/, Annals of Internal Medicine at http://www.annals.org/, and Epidemiology at http://www.epidem.com/). Information on the STROBE Initiative is available at www.strobe-statement.org.
