# Supplementary material for: Osteoprotegerin levels in ST-elevation myocardial infarction: Temporal profile and association with myocardial injury and left ventricular function
Source: PLoS One. 2017 Mar 2;12(3):e0173034. doi: 10.1371/journal.pone.0173034 (PMC5333871; doi:10.1371/journal.pone.0173034)
Supplement: S1 File — (PDF) [file pone.0173034.s002.pdf]

Original Research – Clinical Trial Design

CARDIOLOGY

Cardiology 2010;116:103–109
DOI: 10.1159/000316965

Received: January 12, 2010
Accepted after revision: February 28, 2010
Published online: June 29, 2010

Rationale and Design of the POSTEMI (Postconditioning in ST-Elevation Myocardial Infarction) Study

Shanmuganathan Limalanathan[a, e] Geir Øystein Andersen[a, d, e]
Pavel Hoffmann[b] Nils-Einar Kløw[b] Michael Abdelnoor[c] Jan Eritsland[a, e]

Departments of [a]Cardiology and [b]Radiology, [c]Center for Clinical Research, Unit of Epidemiology and Biostatistics, [d]Center for Clinical Heart Research, Oslo University Hospital Ullevål, and [e]Center for Heart Failure Research, University of Oslo, Oslo, Norway

For editorial comment see p. 101

Key Words
Acute myocardial infarction · Cardiac MRI · Percutaneous coronary intervention · Postconditioning · Reperfusion injury · ST-elevation

Abstract
Rapid reperfusion of the infarct-related coronary artery is essential in the treatment of acute ST-elevation myocardial infarction (STEMI). Paradoxically, restoration of the blood flow to the ischemic area may result in further injury to the myocardium. This phenomenon is described as 'ischemia/reperfusion injury' and the pathophysiological mechanisms are not fully elucidated. A cardioprotective effect of ischemic postconditioning (short repetitive cycles of reperfusion and re-occlusion) has been demonstrated in experimental studies and in pilot studies on patients with acute STEMI treated with primary percutaneous coronary intervention. We present the study design of the Postconditioning in ST-Elevation Myocardial Infarction (POSTEMI) study, which is a prospective, randomized, open-label clinical trial with blinded endpoint evaluation designed to evaluate the effect of postconditioning on final infarct size. Patients with acute STEMI with symptoms of less than 6 h and proximal or mid-coronary artery occlusion will be included. The primary endpoint is infarct size, assessed by cardiac MRI after 4 months. The secondary endpoints are to evaluate the effect of postconditioning on TIMI myocardial perfusion grade, resolution of ST-segment elevation, release of markers of ischemia, left ventricular function and final infarct size related to the area at risk. A total of 260 patients will be included in the study.

Copyright © 2010 S. Karger AG, Basel

Background

In patients suffering from an acute ST-elevation myocardial infarction (STEMI), the occlusion of the infarct-related artery (IRA) is usually caused by plaque rupture and thrombus formation. Rapid reperfusion, either by administration of thrombolytic treatment or primary percutaneous coronary intervention (PCI), is essential in the treatment of acute STEMI [1, 2]. Primary PCI is the preferred therapeutic option if the patient can be referred to a PCI center within the recommended time frame [2]. Restoration of flow in the IRA, as graded by the Thrombolysis In Myocardial Infarction (TIMI) criteria [3], is strongly associated with long-term survival [4]. Reperfu-

KARGER
Fax +41 61 306 12 34
E-Mail karger@karger.ch
www.karger.com

© 2010 S. Karger AG, Basel
0008–6312/10/1162–0103$26.00/0

Accessible online at:
www.karger.com/crd

Dr. Shanmuganathan Limalanathan
Department of Cardiology
Oslo University Hospital Ullevål
NO–0407 Oslo (Norway)
Tel. +47 2211 9100, Fax +47 2211 7399, E-Mail lisq@uus.no

sion of the IRA is a prerequisite, but no guarantee for normalization of myocardial tissue perfusion. The microcirculation can clinically be characterized by variables such as TIMI myocardial perfusion grade (TMPG) [3, 5] and resolution of ST-elevation in ECG [4]. Normalization of these variables early after reperfusion is associated with a favorable clinical outcome [5, 6].

Paradoxically, opening the IRA with re-establishment of flow to the ischemic area may result in further injury of the myocardium and reduce the beneficial effects of reperfusion [7]. This phenomenon has been termed myocardial ischemia/reperfusion (I/R) injury. According to Hearse and Bolli, I/R injury can be categorized into 4 different types of cardiac dysfunction: myocardial stunning, accelerated necrosis, reperfusion induced arrhythmias and lethal reperfusion injury [8]. The pathophysiological mechanisms are not fully elucidated. Oxidative stress, intracellular calcium overload, intracellular acidosis, inflammation and metabolic disturbances are all shown to be involved in the I/R injury. Animal studies have shown that various interventions directed at mediators of I/R injury (e.g. administration of antioxidants, ion-pump inhibitors, anti-inflammatory agents, potassium, insulin-glucose and magnesium) could all substantially reduce the final infarct size [7]. Application of the same principles in clinical studies on patients with acute myocardial infarction, however, has generally been neutral [7]. Evidently, there are large differences between animal models and STEMI patients in a clinical setting, which could account for the lack of efficacy of these therapeutic principles [7, 9]. Identification of the reperfusion injury salvage kinase pathway [9] and mitochondrial permeability transition pore [10, 11] as new targets for cardioprotection has intensified research in this area. Recently, promising results with adjunctive pharmacological treatment aimed at specific targets involved in I/R injury have been reported in STEMI patients [12, 13].

More than 20 years ago, Murry et al. [14] described in an animal model a phenomenon called ischemic preconditioning. Repetitive, short cycles of occlusion-reperfusion of a coronary artery immediately before a longer occlusion resulted in a substantial reduction in final infarct size [14]. As preconditioning must be performed prior to the onset of an acute myocardial infarction, this intervention has not been applicable in the treatment of STEMI, but has been applied in clinical studies of patients undergoing elective cardiac surgery [15].

In 2003, Vinten-Johansen and co-workers first demonstrated the cardioprotective effects of a procedure termed postconditioning. After occlusion of a coronary artery in a canine model, they applied a reperfusion protocol consisting of three short cycles of reperfusion and re-occlusion and were able to demonstrate a reduction of infarct size compared to control animals [16]. Evidence of a clinical benefit of postconditioning is limited. In a smaller study it has been demonstrated that postconditioning resulted in better ST-segment resolution on ECG [17]. In 2005 the first randomized study on postconditioning in patients with acute STEMI was published [18]. In this study on 30 patients, ischemic postconditioning was shown for the first time to reduce the infarct size, measured as creatine kinase release. An improvement of myocardial perfusion grade was also shown [18]. A persistent infarct size reduction was shown to be present at 6 months in a follow-up study on postconditioning in 38 patients [19]. In a meta-analysis reported by Hansen et al., in which six small randomized controlled trials were included comprising a total of 244 patients, a reduction of peak creatine kinase and improvement of left ventricular ejection fraction in favor of the postconditioning group were demonstrated [20].

Very recently, a modest reduction in infarct size, measured by MRI, was demonstrated by a postconditoning protocol applying 30-second intervals of I/R in 86 patients treated with primary PCI. The MRI demonstrated a relative reduction of infarct size in the postconditioning group [21].

Study Rationale

Although pilot studies indicate a beneficial effect of postconditioning in STEMI patients, larger studies in broader STEMI populations are needed to confirm the reported effects on infarct size reduction and further assess the potential clinical benefits. The present study was designed to investigate the effect of postconditioning on infarct size, determined by cardiac MRI, in a larger series of STEMI patients.

The POSTEMI Study

Study Endpoints

The primary objective of the study is to assess the effect of postconditioning on final infarct size in patients with STEMI undergoing primary PCI. Infarct size will be determined by gadolinium late enhancement (LE) cardiac MRI after 4 months.

Table 1. Inclusion and exclusion criteria in the POSTEMI study

Inclusion criteria
– Symptoms of myocardial infarction of less than 6 h combined with ST-segment elevation in ECG, >1 mm in at least two contiguous extremity leads or >2 mm in at least two precordial leads or new left bundle branch block.
– Coronary angiography:
Demonstration of an occluded IRA (TIMI flow grade 0–1):
RCA (segment 1, 2)
LAD (segment 6, 7)
LCX (segment 11).
Immediate demonstration of a normal or near normal flow obtained after the first balloon inflation (TIMI flow grade 2–3).
– Given informed consent.
Exclusion criteria
– Previous myocardial infarction.
– Ongoing treatment of angina pectoris before index infarction.
– Renal failure (creatinine level ≥200 μmol/l).
– Previous aorto-coronary bypass surgery.
– Coronary angiography demonstrating:
Collateral flow to the infarct area;
TIMI flow grade >1 before intervention or <2 after first balloon inflation;
Occlusion of other coronary arteries than IRA.
– An initial strategy of thrombus aspiration decided by operator.
– Thrombolysis given as primary reperfusion treatment.
– Cardiogenic shock, pulmonary congestion or severe hypotension.
– Contraindications to MRI including: cardiac pacemaker/ICD, nerve stimulator, brain aneurysm clips, cochlear implants, claustrophobia.

The secondary objectives are to evaluate the effect of postconditioning on:

- myocardial perfusion grade, using TMPG, at the end of the PCI procedure;
- ST-segment resolution in ECG, 1 h after opening of the IRA;
- peak release of myocardial markers of necrosis;
- left ventricular function and myocardial perfusion by MRI in the acute phase (1–5 days after the infarction);
- final infarct size after 4 months related to the area at risk (evaluated by MRI, acutely and after 4 months), in a subgroup of patients;
- left ventricular function and myocardial perfusion evaluated by MRI after 4 months;
- major adverse cardiovascular events during 1-year follow-up;
- left ventricular function by echocardiographic evaluation in the acute phase and after 4 and 12 months follow-up.

Study Design

The POSTEMI study is a prospective, randomized, single-center, open-label clinical trial with blinded endpoint evaluation where two reperfusion strategies, postconditioning or conventional procedure, will be compared. The study will be performed according to the CONSORT statement [22]. The study population are patients with symptoms consistent with an acute myocardial infarction of less than 6 h duration who fulfill the ECG criteria for primary PCI. The inclusion and exclusion criteria are listed in table 1.

Randomization

The included patients will be randomly allocated to either the postconditioning group or the control group in a 1:1 ratio. The randomization list is generated by computer in a permuted block fashion and transferred to a sequence of sealed, opaque, consecutively numbered envelopes before start of the study. When a patient is considered eligible for the study and has given informed consent, randomization is performed by opening the next envelope in sequence.

Investigational Procedure

All patients will receive aspirin 300 mg, clopidogrel 600 mg and heparin 70 IU/kg (maximum 7,000 IU) before or during procedure and the glycoprotein IIb/IIIa inhibitor eptifibatide during angioplasty, using a standard regimen of weight-adjusted bolus followed by infusion for minimum 12 h [23]. The patients will be treated according to the current guidelines for management of STEMI patients as stated by the European Society of Cardiology [2]. The patients will be randomized to the postconditioning or control group. In the control group, a stent will be implanted immediately after the first balloon inflation and restoration of flow, then the PCI procedure will be completed. In the postconditioning group the IRA will be occluded by balloon inflation after 1 min of reperfusion. The angioplasty balloon will be re-inflated 4 times for 1 min with pressure sufficient to occlude the vessel (fig. 1). Each inflation will be separated by a 1-min interval of reperfusion, according to the procedure described by Staat et al. [18]. The choice of stent type (bare metal or drug-eluting) and dimensions is left to the operator's judgment. Patients will not be included in the trial if an initial strategy of thrombus aspiration has been decided. However, after initial reperfusion and investigational procedure, thrombus aspiration may be applied at the operator's discretion. At the end of the PCI procedure, an intracoronary injection of 200 μg glycerylnitrate will

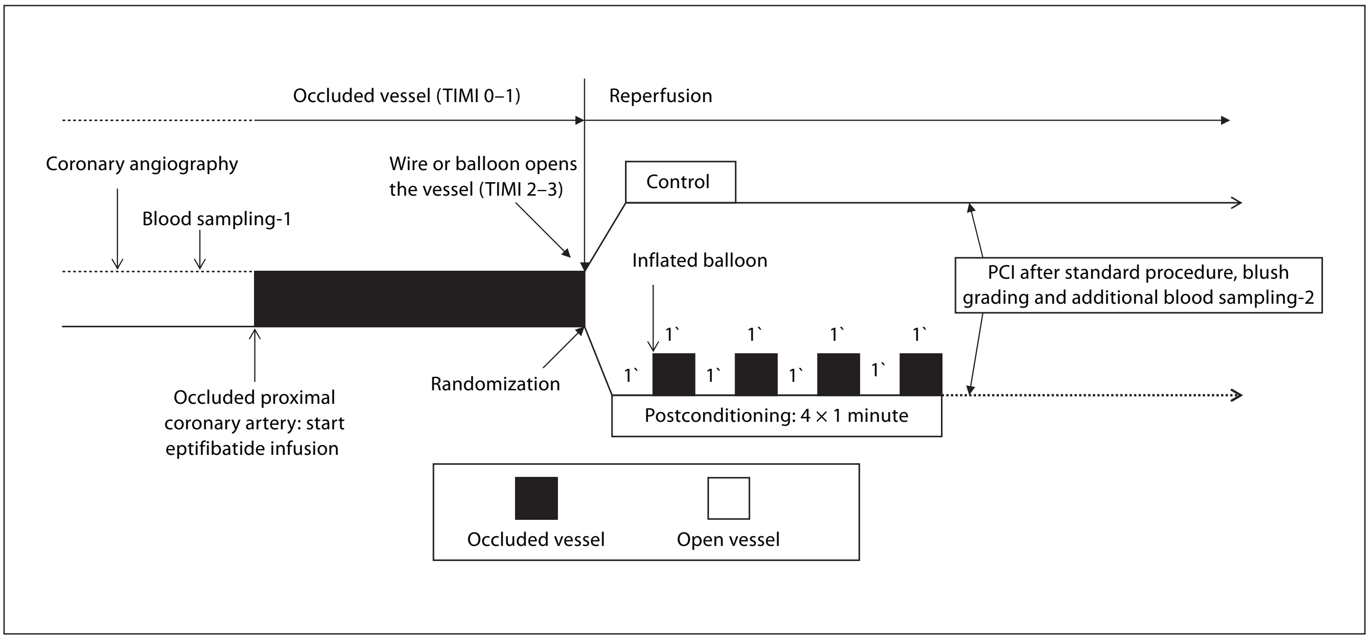

Fig. 1. Flow chart describing the investigational procedure.

be given prior to intracoronary contrast injection in order to assess myocardial perfusion according to TMPG [3, 5], defined according to Gibson et al. [5]:

- grade 0 = failure of contrast to enter the microvasculature (no blush);
- grade 1 = contrast slowly enters but fails to exit the microvasculature;
- grade 2 = delayed entry and exit of contrast from the microvasculature;
- grade 3 = normal entry (blush) and exit of contrast from the microvasculature.

In both groups, blood pressure, heart rate, arrhythmias, oxygen saturation, pain and drugs given during the procedure will be recorded. After the PCI procedure is finished, the patients will be transferred to the cardiac coronary care unit for further observation and treatment. A 12-leads ECG will be taken 1 h after opening of IRA, and ST-segment resolution will be calculated.

MRI Protocol

Two MRI cardiac examinations will be performed, one early (1–5 days) after the STEMI, and one after four months. The early MRI examination will only be performed in a subgroup of patients, depending on the available MRI capacity. MRI is performed on a 1.5-T scanner (Philips Intera, release 11), using five-element synergy-cardiac coil and vector-based ECG. The left ventricle is scanned in two- and four-chamber long axis view using balanced fast field echo sequences for functional analysis, and short axis images are acquired for complete left ventricular volume analysis. T_2-weighted imaging is performed in the cardiac short axis plane using black blood inversion recovery fast spin echo sequences.

First-pass perfusion at rest is performed using ECG triggered fast T_1-enhanced gradient echo technique (TFE) with segmented K-space and saturation prepulse. The LE study is performed 15 min after the first-pass study in short axis view, using 3D TFE technique with inversion prepulses, covering the whole left ventricle. In all patients gadolinium-DTPA (Magnevist, Schering, Germany) is injected using Spectris power injector. For the first-pass perfusion imaging Magnevist 0.05 mmol/kg body weight is injected at the rate of 3 ml/s. For LE imaging, an additional dose of 0.1 mmol/kg of contrast is injected 15 min before acquisition. The scanning parameters are previously described [24].

MRI Analysis

Image analysis is performed on View Forum workstation (Philips Medical Systems). Ejection fraction is calculated by assessment of the volumes of the endocardial contours in diastole and systole of the short axis images.

In diastole, epicardial tracing is also done, to obtain the volume of the left ventricle myocardium. T_2-weighted images are used to quantify the 'area at risk' and to determine the presence of myocardial hemorrhage in the infarcted myocardium. Myocardium with a signal intensity (SI) of more than two standard deviations above the SI in remote non-infarcted myocardium is considered as the 'area at risk'. The area indicated is also manually traced and compared to the area of the whole short axis slice. Myocardial hemorrhage is defined as a dark area in the centre of the 'area at risk'. The hemorrhagic area is manually traced and the area is calculated. On manual tracing of the 'area at risk', the hemorrhagic dark area is included [25, 26]. Myocardial first-pass perfusion is analyzed by measuring the SI versus time. The short axis images are divided into anterior-septal, lateral and inferior-septal wall. SI is always measured in the region of the infarction, selecting the short axis slice considered to be the most representative of the infarction.

Infarct size is assessed on LE images by drawing the contour of the infarcted area in all short axis slices. The volume of the infarction is related to the total left ventricular myocardial volume. Further, short axis slices corresponding to the slices used in the T_2 study are chosen and the hyperintense area (>2 SD above SI in remote, normal myocardium) in these slices is traced. This hyperintense, infarcted area is then related to the area at risk seen at the T_2 imaging.

Microvascular obstruction is defined as the dark area in the center of the hyperintense area in the infarcted myocardium, defined at LE images. The microvascular obstruction is manually traced and the area is calculated. The obstruction is included in manual tracing of the infarcted area.

Blood Samples

Blood will be drawn from the arterial cannula for biobanking and immediate analysis at the end of the procedure. Troponin-T and CKMB will be followed until peak values. Additionally, blood will be sampled for biobanking at the 4 and 12 months' visits. Analysis of biomarkers, including markers of inflammation, hemostasis and brain natriuretic peptide will be performed.

Echocardiography

Left ventricular function, including regional contractility and strain rate imaging, will be assessed by echocardiography during the index hospitalization, at the 4 months' visit and after 1 year.

Table 2. Baseline characteristics of the first 50 randomized patients

Age, years (median, range)	63 (45–86)
Gender	
Male	41 (82%)
Female	9 (18%)
Symptom to balloon time, min	177 (115–265)
Door to balloon time, min	31.5 (26.0–41)
Infarct related artery	
LAD	31 (62%)
LCX	7 (14%)
RCA	12 (24%)
Type of stent implanted	
Bare metal stent	90%
Drug-eluting stent	10%
Left ventricular ejection fraction in acute phase	49.5% (43.5–56.0)

Data are presented as median (25 and 75 percentiles) values or number (percentage).

Clinical Follow-Up

All patients will be followed up with clinical examination, echocardiography and blood analyses during hospitalization and at the 4 and 12 months' visits. All major adverse events during the follow-up period will be recorded.

Statistical Considerations

The primary study endpoint is final infarct size determined by MRI after 4 months, expressed as percentage of left ventricular volume. In a previous series of unselected STEMI patients treated with primary PCI in our institution, infarct size measured by SPECT was 14 ± 8% of left ventricular mass [unpubl. data]. The cardiac MRI method is well correlated with the SPECT method, using radioactive isotope technique [27]. A relative reduction by 20% of infarct size (2.8 percent points) is considered to be of clinical interest. With a 2-sided level of significance of 0.05 and a statistical power of 80%, 127 patients in each group will be needed to show this effect. According to these estimations the study will include 260 patients.

All analyses will be performed according to the intention-to-treat principle. Clinically relevant baseline variables will be compared between the randomized groups. Primary and secondary endpoints will be compared be-

tween the two groups, by χ^2 tests (categorical variables) and by Student's t tests or Mann-Whitney tests when appropriate (continuous variables). A 2-sided p value of <0.05 will be considered statistically significant. Multivariate analyses to control for confounding effect of different variables will be performed [28]. All analyses will be performed by widely accepted statistical and epidemiological software.

Study Approval and Progression

The study will be conducted in accordance with the ethical principles of the Declaration of Helsinki as adopted by the 18th World Medical Assembly in Helsinki 1964 and subsequent versions. The protocol has been approved by the Regional Committee for Medical Research Ethics and is registered at the ClinicalTrials.gov (Study ID No. Po 1506). Oslo University Hospital is responsible for the study. All serious adverse events will be immediately reported to an independent data and safety monitoring board. The POSTEMI study is funded by the Norwegian Health and Rehabilitation Foundation. The patient inclusion in the POSTEMI study started in spring 2009 with an estimated inclusion period of about 2 years. Some baseline variables of the first 50 included patients are given in table 2.

Clinical Implications

In acute STEMI, current guidelines emphasize timely revascularization and adjunctive medical treatment, including anti-thrombotics [2]. Adherence to the guidelines has been shown to improve the prognosis of STEMI patients [29]. Nevertheless, a number of patients suffer large infarctions with a subsequent increased risk of heart failure or death. Thus, additional measures to reduce myocardial damage in STEMI have been pursued. Therapeutic strategies to demonstrate a reduction of I/R injury have probably been hampered by the lack of sensitive methods to measure final infarct size and in particular, myocardial salvage. The present study may contribute to establish the role of cardiac MRI in the determination of the area at risk and final infarct size. Targeting novel intracellular signaling mechanisms of I/R injury in STEMI by pharmacological or ischemic postconditioning has shown promising results, which have to be confirmed. Further evaluation of the effect of postconditioning in larger groups of STEMI patients is needed. If a reduction of final infarct size can be unequivocally shown by this procedure, larger studies with clinical endpoints may be warranted.

Appendix

Data and safety monitoring board: Dan Atar, MD, PhD, Department of Cardiology, Oslo University Hospital Aker/Ullevål, and Bjørn Bendz, MD, PhD, Department of Cardiology, Oslo University Hospital Gaustad.

References

1 Fibrinolytic Therapy Trialists (FTT) Collaborative Group: Indications for fibrinolytic therapy in suspected acute myocardial infarction: collaborative overview of early mortality and major morbidity results from all randomised trials of more than 100 patients. Lancet 1994;343:311–322.

2 Van de Werf F, Bax J, Betriu A, et al: Management of acute myocardial infarction in patients presenting with persistent ST-segment elevation. Eur Heart J 2008;29:2909–2945.

3 TIMI Study Group: Definitions used in TIMI trials. http://www.timi.org.

4 Ndrepa G, Mehilli J, Schulz S, et al: Prognostic significance of epicardial blood flow before and after percutaneous coronary intervention in patients with acute coronary syndromes. J Am Coll Cardiol 2008;52:512–517.

5 Gibson CM, Cannon CP, Murphy SA, et al: Relationship of TIMI myocardial perfusion grade to mortality after administration of thrombolytic drugs. Circulation 2000;101:125–130.

6 de Lemos JA, Braunwald E: ST segment resolution as a tool for assessing the efficacy of reperfusion therapy. J Am Coll Cardiol 2001;38:1283–1294.

7 Yellon DM, Hausenloy DJ: Myocardial reperfusion injury. N Engl J Med 2007;357:1121–1135.

8 Hearse DJ, Bolli R: Reperfusion induced injury: manifestations, mechanisms, and clinical relevance. Cardiovasc Res 1992;26:101–108.

9 Hausenloy DJ, Yellon DM: New directions for protecting the heart against ischemia-reperfusion injury: targeting the Reperfusion Injury Salvage Kinase (RISK) pathway. Cardiovasc Res 2004;61:448–460.

10 Hausenloy DJ, Yellon DM: The mitochondrial permeability transition pore: its fundamental role in mediating cell death during ischemia and reperfusion. J Mol Cell Cardiol 2003;35:339–341.

11 Hausenloy DJ, Duchen MR, Yellon DM: Inhibiting mitochondrial permeability transition pore opening at reperfusion protects against ischemia reperfusion injury. Cardiovasc Res 2003;60:617–625.

12 Piot C, Croisille P, Staat P, et al: Effect of cyclosporine on reperfusion injury in acute myocardial infarction. N Engl J Med 2008;359:473–481.

13 Atar D, Petzelbauer P, Schwitter J, et al: Effect of intravenous FX06 as an adjunct to primary percutaneous coronary intervention for acute ST-segment elevation myocardial infarction. J Am Coll Cardiol 2009;53:720–729.

14 Murry CE, Jennings RB, Reimer KA: Preconditioning with ischemia: a delay of lethal injury in ischemic myocardium. Circulation 1986;74:1124–1136.
15 Granfeldt A, Lefer DJ, Vinten-Johansen J: Protective ischemia in patients: preconditioning and postconditioning. Cardiovasc Res 2009;83:234–246.
16 Zhao ZQ, Corvera JS, Halkos ME, et al: Inhibition of myocardial injury by ischemic postconditioning during reperfusion: comparison with ischemic preconditioning. Am J Physiol Heart Circ Physiol 2003;285:H579–H588.
17 Laskey WK: Brief repetitive balloon occlusions enhance reperfusion during percutaneous coronary intervention for acute myocardial infarction: a pilot study. Cathet Cardiovasc Interven 2005;65:361–367.
18 Staat P, Rifoul G, Piot C, et al: Postconditioning the human heart. Circulation 2005;112:2143–2148.
19 Thibault H, Piot C, Staat P, et al: Long-term benefit of postconditioning. Circulation 2008;117:1037–1044.
20 Hansen P, Thibault H, Abdulla J: Postconditioning during primary percutaneous coronary intervention: a review and meta-analysis. Int J Cardiol 2009, E-pub ahead of print.
21 Lønborg J, Kelbæk H, et al: Cardioprotective effects of ischemic postconditioning in patients treated with primary percutaneous coronary intervention, evaluated by magnetic resonance. Circ Cardiovasc Interv 2010;3:34–41.
22 Moher D, Schulz KF, Altman DG; for the CONSORT Group: The CONSORT statement: revised recommendations for improving the quality of reports of parallel-group randomised trials. Lancet 2001;357:1191–1194.
23 Gurm HS, Smith DE, Collins JS, et al: The relative safety and efficacy of abciximab and eptifibatide in patients undergoing primary percutaneous coronary intervention. J Am Coll Cardiol 2008;51:529–535.
24 Hoffmann P, Halvorsen S, Stensaeth KH, et al: Myocardial perfusion in ST-elevation myocardial infarction treated successfully with primary angioplasty. Scand Cardiovasc J 2006;40:96–104.
25 Ganame J, Messalli G, Dymarkowski S, et al: Impact of myocardial haemorrhage on left ventricular function and remodelling in patients with reperfused acute myocardial infarction. Eur Heart J 2009;30:1440–1449.
26 O'Regan DP, Ahmed R, Karunanithy N, et al: Reperfusion hemorrhage following acute myocardial infarction: assessment with T2* mapping and effect on measuring the area at risk. Radiology 2009;250:916–922.
27 Carlsson M, Ubachs JFA, Hedström E, Heiberg E, Jovinge S, Arheden H: Myocardium at risk after acute infarction in humans on cardiac magnetic resonance. J Am Coll Cardiol Img 2009;2:569–576.
28 Pocock SJ: Clinical Trials: A Practical Approach. Chichester, John Wiley and Sons, 1983.
29 Kalla K, Christ G, Karnik R, et al: Implementation of guidelines improves the standard of care: the Viennese registry on reperfusion strategies in ST-elevation myocardial infarction (Vienna STEMI Registry). Circulation 2006;113:2398–2405.
