# Supplementary material for: Osteoprotegerin levels in ST-elevation myocardial infarction: Temporal profile and association with myocardial injury and left ventricular function
Source: PLoS One. 2017 Mar 2;12(3):e0173034. doi: 10.1371/journal.pone.0173034 (PMC5333871; doi:10.1371/journal.pone.0173034)
Supplement: S1 Protocol — (PDF) [file pone.0173034.s004.pdf]

Clinical Trial Protocol

Postconditioning in ST-elevation myocardial infarction treated with primary PCI

A prospective, randomized, single center, open-label clinical trial with blinded primary endpoint evaluation

The POSTEMI Study Group

1. Introduction

Reperfusion therapy, administered as early as possible after start of symptoms, has substantially improved the prognosis in acute ST-elevation myocardial infarction. Still, however, many patients suffer large infarctions, subsequently with an increased risk of heart failure, arrhythmias, and death. Experimental studies in animals have shown that the reperfusion *per se* may trigger intracellular pathways which in the end may result in further damage to the myocardium. These mechanisms, which to a large extent are elucidated, have collectively been termed ischemia-reperfusion injury pathways. Inhibition of these injury mechanisms could potentially reduce the extent of the final myocardial infarction and thus improve the long-term prognosis, but so far intervention studies with pharmacological inhibitors have not shown effects. Recently, inhibition of the injury pathways by mechanical intervention has been suggested as an alternative therapeutic approach in patients with myocardial infarction undergoing catheter-based revascularization.

2. Background

2.1 Myocardial reperfusion injury

Reperfusion therapy, started as early as possible after debut of symptoms, is essential in the treatment of acute ST-elevation myocardial infarction (STEMI) (1). When given early after start of symptoms, thrombolytic treatment has proved to be effective (2) and in the first 3 hours is probably as effective as primary PCI (3). However, provided a time delay <90 minutes from diagnosis to treatment, primary PCI is overall a better alternative (3-5). With

primary PCI TIMI-flow grade 2 or 3 (6) in the infarct related artery (IRA) can be attained in 95% of the patients (7,8).

Establishment of a normal TIMI-flow in IRA is a necessary condition, but no guarantee, for normalization of tissue perfusion. Myocardial blush grade (9) and regression of ST-elevation (10) are variables which can be used clinically to characterize the circulation at the tissue level after reperfusion of STEMI. Normalization of these variables has been shown to be associated with improved clinical outcomes (9,10). Paradoxically, opening of an occluded IRA can result in further injury at the microcirculatory level, thus reducing the beneficial effect of reperfusion therapy, a process termed myocardial reperfusion injury. Endothelial cells and cardiomyocytes not irreversibly damaged by ischemia can be further injured by the reperfusion and undergo cell death, either by necrosis or by apoptosis (11).

Myocardial reperfusion injury causes different types of cardiac dysfunction and the pathophysiological mechanisms are only partly elucidated. According to Yellon and Hausenloy (11) four types of cardiac dysfunction can be characterized: Myocardial stunning, the no-reflow phenomenon, reperfusion arrhythmias, and lethal reperfusion injury. The mediators of injury include reactive oxygen species, intracellular calcium overload, pH perturbations, and inflammatory and metabolic mediators. Experimentally, it has been shown that various interventions directed at mediators of reperfusion injury can reduce the infarction damage up to 50% (11).

The complex mechanisms behind the myocardial reperfusion injury open for a multitude of therapeutic interventions. In experimental animal models treatment with antioxidants, ion pump inhibitors, anti-inflammatory principles, and metabolic modulation with potassium, insulin, and glucose, as well as magnesium, all have proved efficacious in reducing myocardial damage. Clinical studies in patients with acute myocardial infarction applying the same intervention principles, however, have generally been neutral (11).
Evidently, there are large differences between animals in a controlled experimental situation and patients with an acute myocardial infarction, which can account for the lack of efficacy of promising therapeutic principles in the latter setting (11). So far, optimalization of reperfusion in STEMI includes measures to shorten the ischemic period as much as possible, administration of proper antithrombotic treatment, to give nitroglycerine when spasms occur, and in selective cases apply mechanical devices to remove thrombus.

2.2 Postconditioning

In 2003 Zhao et al in a canine model were able to show that short occlusions (30 seconds) given intermittently 3 times with 30 seconds intervals, starting 30 seconds after opening of an LAD which had been occluded for 60 minutes, resulted in a reduction of infarct size from 25% (control group, without further occlusions) to 14% of the left ventricle after 3 hours of reperfusion. They termed this intervention ischemic postconditioning and also showed that the effect of postconditioning was comparable to the effect of preconditioning (12).
Preconditioning implied a transient occlusion of the vessel (here for 5 minutes), reperfusion (10 minutes) and then occlusion for 60 minutes before a new reperfusion period of 3 hours. The effect of preconditioning had been reported previously (13) and has to large extent been studied in different experimental models. The mechanisms behind the two infarct-reducing principles are assumed, at least partly, to be dissimilar. The effect of postconditioning on infarct size has been confirmed in various animal models (14). The postcondioning procedure

has been shown to reduce oxidative stress, decrease intracellular calcium overload, attenuate apoptosis, reduce neutrophil accumulation, and modify pH changes. Also, “reperfusion injury salvage kinase (RISK)-pathway” is activated and mitochondrial “permeability transition pore (PTP)” is inhibited, contributing to a reduced myocardial reperfusion injury (11).

2.3 Postconditioning in STEMI

Evidence of a clinically beneficial effect of postconditioning in STEMI is limited. In a retrospective study Darling et al (15) reported that patients requiring four or more balloon inflations during primary PCI in STEMI (n=56) had a lower peak CK release than patients with 1-3 balloon inflations (n=59). Laskey (16) randomized STEMI patients to an additional 90 seconds period of balloon inflation (n=10) or not (n=7) after opening of the IRA and a reperfusion period of 3-5 minutes. The postconditioning group had better ST-segment resolution in ECG and a higher coronary flow velocity reserve measured by Doppler method than the control group. In a randomized study in STEMI patients Ma et al (17) found higher coronary blood flow velocity after postconditoning compared with the control group and after 8 weeks greater improvement of echocardiographic wall-motion score index in the postcoditoning group than in the control group. Also, peak release of CK and CK-MB were reduced in the postconditioning group.

In a study of 30 STEMI patients treated with primary PCI, Staat et al (18) randomized the patients to a postconditioning protocol or a control group, undergoing regular PCI. All patients were treated with the direct stenting technique. Postconditioning was given by 4 new inflations, each for 1 minute, separated by 1 minute of reperfusion, starting 1 minute after opening of IRA. The procedure was well tolerated in all patients and no adverse events occurred. CK release measured by area under the curve was significantly reduced and myocardial blushing grade significantly improved in the postconditioning group. In a new study the same research group randomized 38 STEMI patients according to the same treatment protocol (19). In this study infarct size was assessed after 6 months (SPECT method) and after 1 year (echocardiography). Infarct size was significantly less in the postconditioning group compared with the control group after 6 months. After 1 year wall-motion score index and ejection fraction were significantly better in the postconditioning group compared with the control group.

Thus, pilot trials in selected STEMI patients indicate a beneficial effect of postconditioning on infarct size and on selected variables of reperfusion. Larger studies in a real-world setting are needed to assess whether this principle can have effects considered to be of clinical importance in STEMI patients.

3. Aims of the study

3.1 Study objectives

The primary objective of the trial is to assess the effect of postconditoning in STEMI patients undergoing primary PCI on final infarct size, evaluated by magnetic resonance imaging (MRI) after 4 months.

Secondary objectives are to evaluate the effect of postconditioning on myocardial blushing at the end of the PCI procedure and the effect on ECG ST-segment resolution 1 hour after opening of IRA. Peak release of myocardial markers troponin-T and CK-MB will be compared between the treatment groups. The incidence of serious arrhythmias and signs of heart failure will be recorded. The patients will be followed up for 1 year and clinical cardiovascular events during follow-up will be noted. Infarct size and left ventricular function will be evaluated by echocardiography at 1 year. In the acute stage blood samples for biobanking will be taken and analyzed at a later stage with respect to prognostic factors.

3.2 End points

Primary end point:

Infarct size, assessed after 4 months with MRI and expressed as percentage of left ventricular mass.

Secondary end points:

Myocardial blushing grade, assessed at the end of PCI procedure
ST-resolution in ECG, assessed after 1 hour in the ECG-lead with the greatest deviation at baseline.
Troponin-T and CK-MB peak release values
Echocardiographic evaluation of left ventricular function, assessed after 1 year
Incidence of treated arrhythmias and heart failure during initial hospitalization
Incidence of death, non-fatal myocardial infarction, unstable angina, heart failure, and cerebrovascular disease during 1-year follow up.
In a subgroup of patients, an early (within 2 days) MRI study will be performed, in order to assess the “area at risk” (20). Due to limitations of capacity, this early MRI study will depend on the day-to-day availability of MRI. Thus, only a subgroup will undergo an early MRI. Also, depending on the capacity, an early echo-study with evaluation of left ventricular function will be performed in a subgroup of patients.

4 Design / Methods

4.1 Treatment comparisons

STEMI patients undergoing primary PCI and fulfilling inclusion criteria will after giving informed consent to participate be randomized to a postconditioning group or a control group. After opening of IRA and establishment of TIMI-flow grade 2 or 3, the control group continues the procedure with stenting. In the postconditioning group 4 additional balloon inflations separated by 1 minute reperfusion are given, starting after 1 minute of reperfusion. After this the procedure will continue with stenting as usual. All routine treatment for primary PCI will be given as usual to both groups, during the acute procedure and in the follow-up period.

4.2 Patient population

The source population are patients with symptoms consistent with myocardial infarction lasting less than 6 hours combined with ST-segment elevation in ECG, ≥1 mm in at least 2

standard leads or ≥2 mm in at least 2 precordial leads. Patients without previous angina pectoris and no previous myocardial infarction will be possible candidates for the study. Exclusion criteria will be any contraindication to MRI, chest pain of more than 6 hours duration, thrombolytic treatment prior to PCI, or cardiogenic shock.

Only patients with single vessel occlusion (TIMI-flow 0-1 prior to PCI, with no retrograde filling) in proximal or mid RCA (segment 1,2), proximal or mid LAD (segment 6,7), or proximal CX (segment 11) will be asked to participate. Further, restoration of TIMI-flow 2-3 in IRA following the first balloon inflation will be required before randomization.

The patient must give informed consent to participate in the study before randomization. Patients who already participate in a randomized study are not eligible for the present study.

4.3 Procedures

Randomization
The included patients will be randomly allocated to the postconditioning group or to the control group. The randomization list will be generated by computer and transferred to a sequence of sealed, numbered envelopes by epidemiologist/statistician before start of the study. When a patient is considered eligible for the study and has given informed consent, randomization is performed by opening the next envelope in the sequence.

Coronary angiography
All patients will receive aspirin, clopidogrel, and heparin before or during angioplasty. The glycoprotein IIb/IIIa inhibitor abciximab (ReoPro®) will be given to all patients during the angioplasty, using a standard regimen of weight-adjusted bolus followed by infusion for 12 hours (21). Visualization of the coronary arteries will be performed according to standard procedures.

Postconditioning protocol
Following angiography and first balloon inflation and after informed consent, eligible patients will be randomized to either control or to the postconditioning group. In the postconditioning group, starting after 1 minute of reflow the angioplasty balloon will be reinflated 4 times for 1 minute, with pressure sufficient to occlude the artery. Each reinflation will be separated by 1 minute of reflow, as described by Staat et al (18). After the postconditioning a stent will be implanted and the angioplasty procedure will be completed according to each operators judgement. Any additional balloon inflations will be recorded. In the control group a stent will be implanted immediately after reflow and the PCI procedure will be completed. All additional dilations will be recorded.

In both groups TIMI flow, ST – segment resolution, and reperfusion arrhythmias will be assessed.

TIMI myocardial perfusion (TMP) grade
TMP grade will be assessed at the end of the PCI procedure, according to previously described methods (22,23). The angiography will be done following intracoronary injection of 200 μg glycerol nitrate, using the contrast medium Ioversol 350 mg I/ml (Optiray, Tyco

Healthcare). TMP grading will be done by two observers independently. TMP grades will be defined according to the method of Gibson et al (23)

Regression of ST-segment deviation
The ECG lead with the largest ST deviation will be used for analysis. ST deviation 1 hour after opening of IRA will be compared with the pre-PCI value and percent regression will be calculated.

MRI protocol
All patients will have a cardiac MRI examination 4 months after the infarction. A subgroup of the patients will also be examined within 2 days of the infarction, and again after 4 months. Cardiac MRI will be performed on a 1.5 T whole body scanner (Philips Intera, Best, the Netherlands), using five element synergy-cardiac coil and vector-based ECG. The MRI studies will be performed following conventional methodology (24-27), as summarized in Hoffmann et al (28).

4.4 Sample size

The primary study end point is final infarct size, measured after 4 months with the MRI method, and given as a percentage of left ventricular volume. In this respect, the MRI method is well correlated with the SPECT method, using radioactive isotope technique (29). In a previous series of STEMI patients undergoing PCI and followed up with SPECT after 3 months (n = 200), we measured a mean infarct size of 14 ± 8% of left ventricular mass (Andersen GØ, unpublished data). A relative reduction by 20% of infarct size would be considered to be of clinical interest. With a 2-sided level of significance 0.05 and a statistical power of 80%, 127 patients in each group will be needed to show this effect. According to these estimations the study will include 260 patients.

4.5 Statistical analysis

All analyses will be performed according to the intention-to-treat principle. Clinically relevant baseline variables will be compared between the randomized groups. Regarding outcome, primary and secondary endpoints will be compared between the two groups. Categorical variables will be compared by chi-square tests and continuous variables by t-tests. A 2-sided p-value <0.05 will be considered statistical significant. No adjustments will be made for multiple comparisons.

In secondary analyses adjustments for unequalities in baseline characteristics or possible confounders will be undertaken.

All analyses will be performed using widely accepted statistical / epidemiological software.

4.6 Ethics
The study will be conducted in accordance with the ethical principles of the Declaration of Helsinki as adopted by the 18th World Medical Assembly in Helsinki 1964 and subsequent

versions. The trial has been approved by national and institutional regulatory authorities, including the Regional Ethics Committee.

All patients will be treated according to the current guidelines for management of STEMI patients by the European Society of Cardiology (30). In the clinical studies which so far have been published (16-19), no untoward effect by the postconditioning procedure has been recorded.

4.7. Steering committee

Jan Eritsland, MD, PhD	Coronary Care Unit, Dept. of Cardiology
Geir Øystein Andersen, MD, PhD	Coronary Care Unit, Dept. of Cardiology
Pavel Hoffmann, MD, PhD	Dept. of Cardiovascular Radiology
Nils-Einar Kløw, MD, PhD	Dept. of Cardiovascular Radiology

Biobanking will be performed in collaboration with
Ingebjørg Seljeflot, PhD Center for Clinical Heart Research

all at Ullevål University Hospital, Oslo, Norway.

4.8 Schedule

July 2008: Study approved by Datatilsynet and Regional Ethics Committee
Jan 2009: Start patient inclusion
2011: Publication of main results

References

1. De Luca G, Suryapranata H, Ottervanger JP, Antman EM. Time delay to treatment and mortality in primary angioplasty for acute myocardial infarction. Circulation 2004;109:1223-5.
2. Fibrinolytic Therapy Trialists´ (FTT) Collaborative Group. Indications for fibrinolytic therapy in suspected acute myocardial infarction: collaborative overview of early mortality and major morbidity results from all randomised trials of more than 1000 patients. Lancet 1994;343:311-22.
3. Zijlstra F, Jones PM, Grines CL et al. Clinical characteristics and outcome of patients with early (<2 h), intermediate (2-4 h) and late (>4 h) presentation treated by primary coronary angioplasty or thrombolytic therapy for acute myocardial infarction. Eur Heart J 2002;23:550-7.
4. Keeley EC, Boura JA, Grines CL. Primary angioplasty versus intravenous thrombolytic therapy for acute myocardial infarction: a quantitative review of 23 randomised trials. Lancet 2003;361:13-20.
5. Nallamothu BK, Bradley EH, Krumholz HM. Time to treatment in primary percutaneous coronary intervention. N Engl J Med 2007;357:1631-8.
6. Mangschau A, Bendz B, Eritsland J et al. Ett hundre pasienter behandlet med primær angioplastikk ved akutt hjerteinfarkt. Tidsskr Nor Lægeforen 2001;121:775-9.

7. Eritsland J, Kløw N-E, Westheim A, Bendz B, Mangschau A. Primær angioplastikk ved akutt ST-hevningsinfarkt hos eldre. Tidsskr Nor Lægeforen 2005;125:2922-4.
8. Gibson CM, Cannon CP, Murphy SA et al. Relationship of the TIMI myocardial perfusion grades, flow grades, frame count, and percutaneous coronary intervention to long-term outcomes after thrombolytic administration in acute myocardial infarction. Circulation 2002;105:1909-13.
9. de Lemos JA, Braunwald E. ST segment resolution as a tool for assessing the efficacy of reperfusion therapy. J Am Coll Cardiol 2001;38:1283-94.
10. Piper HM, García-Dorado D, Ovize M. A fresh look at reperfusion injury. Cardiovasc Res 1998;38:291-300.
11. Yellon DM, Hausenloy DJ. Myocardial reperfusion injury. N Engl J Med 2007;357:1121-35.
12. Zhao Z-Q, Corvera JS, Halkos ME et al. Inhibition of myocardial injury by ischemic postconditioning during reperfusion: comparison with ischemic preconditioning. Am J Physiol Heart Circ Physiol 2003;285:H579-88.
13. Murry CE, Jennings RB, Reimer KA. Preconditioning with ischemia: a delay of lethal cell injury in ischemic myocardium. Circulation 1986;74:1124-36.
14. Tissier R, Berdeaux A, Ghaleh B, et al. Making the heart resistant to infarction: how can we further decrease infarct size? Front Bioscience 2008;13:284-301.
15. Darling CE, Solari PB, Smith CS, et al. "Postconditioning" the human heart: Multiple balloon inflations during primary angioplasty may confer cardioprotection. Basic Res Cardiol 2007;102:274-8.
16. Laskey WK. Brief repetitive balloon occlusions enhance reperfusion during percutaneous coronary intervention for acute myocardial infarction: A pilot study. Cathet Cardiovasc Interven 2005;65:361-7.
17. Ma X, Zhang X, Li C, Luo M. Effect of postconditioning on coronary blood flow velocity and endothelial function and LV recovery after myocardial infarction. J Interven Cardiol 2006;19:367-75.
18. Staat P, Rifoul G, Piot C et al. Postconditioning the human heart. Circulation 2005;112:2143-8.
19. Thibault H, Piot C, Staat P et al. Long-term benefit of postconditioning. Circulation 2008;117:1037-44.
20. Friedrich MG, Abdel-Aty H, Taylor A, et al. The salvaged area at risk in reperfused acute myocardial infarction as visualized by cardiovascular magnetic resonance. JACC 2008;51:1581-7.
21. Montalescot G, Antoniucci D, Kastrati A, et al. Abciximab in primary stenting of ST-elevation myocardial infarction: a European meta-analysis on individual patients´data with long-term follow-up. Eur Heart J 2007;28:443-9.
22. van`t Hof AWJ, Liem A, Suryapranata H, Hoorntje JC, de Boer MJ, Zijlstra F. Angiographic assessment of myocardial reperfusion in patients treated with primary angioplasty for acute myocardial infarction. Myocardial blush grade. Circulation. 1998;97:2302-6.
23. Gibson CM, Cannon CP, Murphy SA, et al. Relationship of TIMI myocardial perfusion grade to mortality after administration of thrombolytic drugs. Circulation. 2000;101:125-30.
24. Aletras AH, Gauri ST, Natanzon A, et al. Retrospective determination of the area at risk for reperfused acute myocardial infarction with T2-weighted cardiac magnetic resonance imaging. Circulation 2006;113:1865–70.

25. Stork A, Muellerleile K, Bansmann PM, et al. Value of T2-weighted, first-pass and delayed enhancement, and cine CMR to differentiate between acute and chronic myocardial infarction. European Radiology 2007;17:610–7.
26. Nagel E, Klein C, Paetsch I, et al. Magnetic resonance perfusion measurements for the noninvasive detection of coronary artery disease. Circulation 2003;108:432-7.
27. Klein C, Nekolla SG, Bengel FM, et al. Assessment of myocardial viability with contrast-enhanced magnetic resonance imaging. Comparison with positron emission tomography. Circulation 2002;105:162-7.
28. Hoffmann P, Halvorsen S, Stensæth K-H, et al. Myocardial perfusion in ST-elevation myocardial infarction treated successfully with primary angioplasty. Scand Cardiovasc J 2006;40:96-104.
29. Lunde K, Solheim S, Aakhus S, et al. Intracoronary injection of mononuclear bone marrow cells in acute myocardial infarctin. N Engl J Med 2006;355:1199-209.
30. Van de Werf F, Ardissino D, Betriu A, et al. Management of acute myocardial infarction in patients presenting with ST-segment elevation. Eur Heart J 2003;24:28-66.
